# Standardized clinical assessments and advanced AI-driven instruments used to evaluate neurofunctional deficits, including within biomarker based framework, in Parkinson’s disease - human intelligence made vs. AI models - systematic review

**DOI:** 10.3389/fmed.2025.1565275

**Published:** 2025-06-13

**Authors:** Aurelian Anghelescu, Constantin Munteanu, Aura Spinu, Vlad Ciobanu, Cristina Popescu, Ioana Elena Cioca, Ioana Andone, Simona-Isabelle Stoica, Mihaela Mandu, Ana Rebedea, Sebastian Giuvara, Alin-Daniel Malaelea, Andreea-Iulia Vladulescu-Trandafir, Maria-Veronica Morcov, Gelu Onose

**Affiliations:** ^1^Department of Specific Discipline, Faculty of Midwifery and Nursing, University of Medicine and Pharmacy “Carol Davila”, Bucharest, Romania; ^2^Teaching Emergency Hospital “Bagdasar-Arseni”, Bucharest, Romania; ^3^Department of Biomedical Sciences, Faculty of Medical Bioengineering, University of Medicine and Pharmacy “Grigore T. Popa”, Iaşi, Romania; ^4^Department of Clinical Education, Physical and Rehabilitation Medicine, Faculty of Medicine, University of Medicine and Pharmacy “Carol Davila”, Bucharest, Romania; ^5^Department of Computer Science, Politehnica University of Bucharest, Bucharest, Romania; ^6^Department of Basic Medical Sciences, Faculty of Midwifery and Nursing, University of Medicine and Pharmacy “Carol Davila”, Bucharest, Romania; ^7^National Teaching Centre for Neuro-psycho-motor Rehabilitation in Children “Dr. N. Robanescu”, Bucharest, Romania

**Keywords:** Parkinson’s disease, human intelligence, artificial intelligence, assessment instruments, ICF framing, ChatGPT4.o, ChatGPT Scholar

## Abstract

**Introduction:**

Considering the extensive development of artificial intelligence (AI) facilities, like Generative Pre-Trained Transformer (ChatGPT) 4.o and ChatGPT Scholar, we explored their abilities to conduct a systematic literature review. Using as a specific domain, an attempt to frame/methodize clinical assessment instruments used to evaluate neuro-functional deficits in Parkinson’s disease (PD) – including framed through the ICF(-DH) paradigm – for the above-mentioned comparison between human intelligence (HI) and AI, this paper is as well, a follow-up regarding the most actual subject matter of the AI’s capabilities evolution in this respect. As well-known clinical-/paraclinical-/functional evaluations, using assessment quantitative (as much as possible) instruments, are basic endeavors for rehabilitation, as they enable setting of appropriate and realistic therapeutic-rehabilitative specific goals.

**Methods:**

Within the actual work, we have first achieved a narrative synthesis of the main molecular mechanisms involved in PD pathophysiology, underpinning its clinical appearance and evolution. To fundament our knowledge on an up-to-date information regarding the clinical-functional evaluation tools practiced in PD, we systematically reviewed the literature in this domain, published in the last 6 years, through a PRISMA type method for filtering/selecting the related bibliographic resources. The same keywords combinations/syntaxes have been used contextually, also to dialogize with ChatGPT4.o and ChatGPT.

**Results:**

Scholar Applying PRISMA type methodology (HI achieved), we have selected, matching the filtering criteria, 24 articles. Interrogating the two AI above-mentioned models, we obtained quite difficult to be availed/useful – comparative to our HI obtained – outcomes. Thus, when interrogating ChatGPT4.o, ChatGPT Scholar repeatedly, they provided - partially diverse - inappropriate related answers, including ones pending on the interrogator’s IP, although they claimed to have this capacity.

**Discussion:**

We consider, regarding their capabilities to achieve systematic literature reviews, that neither ChatGPT 4.o nor ChatGPT Scholar still cannot succeed this (yet, they keep improving lately). Additionally, we have consistently extended, including within a narrative related literature review, our ‘dialogue” with these two AI facilities regarding their availability to enhance the related evaluation instruments accuracy on neurofunctional assessments within biomarker-based frameworks. So, our research aimed basically to emphasize the main topical data regarding these two important paradigms of knowledge (based on HI and on AI) acquirements – considering the impetuous development of the latter – and thus, possibly to contribute inclusively at improving the actual performances to achieve Systematic Literature Reviews through the PRISMA type method – for the moment still better served by HI.

## 1 Introduction

Parkinson’s disease (PD) is a chronic progressive and extensive degenerative disease of the extrapyramidal nervous system that can cause motor symptoms (tremor, muscle rigidity, bradykinesia/slow movements, and postural instability) as well as non-motor symptoms (neuro-vegetative, psycho-cognitive/dementia, pain/sensory, and/or sleep disturbances) (1–3).

Parkinson’s disease is a multisystem disorder, a consequence of multi-neuropeptidergic dysfunction, which includes dopaminergic, serotoninergic, cholinergic, and noradrenergic systems. This multipath way disturbance leads, including, to the expression of the non-motor symptoms, recognized as being integrated into the concept of PD, and preceding motor signs (4).

Clinical peculiarities raise suspicion of different genetic (see further) and etiological influences, with distinct path-physiological mechanisms including as regards neuro-transmitter systems, with particular consequent aspects, also, of functional imaging (4, 5).

Hence, the modern paradigm of PD would be, in short: Parkinsonian patients constitute a heterogeneous nosology group, presenting a wide range of motor and non-motor symptoms that progress over time and differ among individuals (5).

**FIGURE 1:**
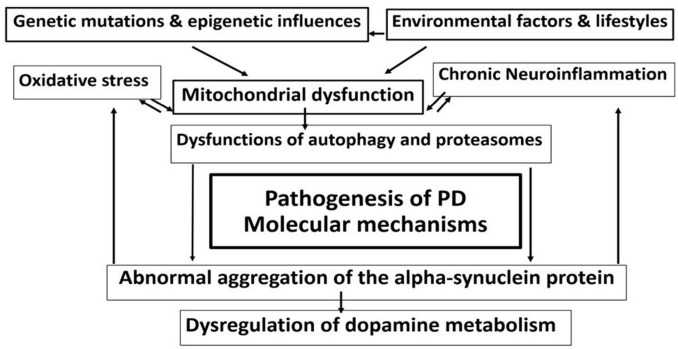
Complex intermingled molecular mechanisms in the pathophysiology of Parkinson’s disease (PD) create an interconnected harmful cycle, leading to progressive neuronal loss and clinical aggravation.

**FIGURE 2:**
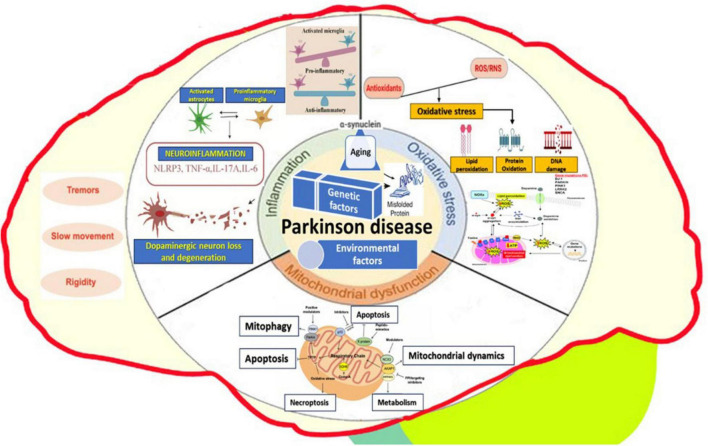
Complex cellular mechanisms involved in Parkinson’s disease - altered gene expression, mitochondrial dysfunction, oxidative stress, protein aggregation, neuroinflammation, and external environmental factors.

Several complex cellular mechanisms create a vicious cycle of dysfunctions and con-tribute to the neurodegenerative development of Parkinson’s disease (Figure 1). These mechanisms, which include altered gene expression, mitochondrial dysfunction, oxidative stress, calcium dysregulation, protein aggregation, and chronic neuroinflammation (Figure 2), are crucial in understanding the progression of the disease (3).

One of the key elements is altered gene expression (6). Dysregulation of genes involved in dopamine synthesis and metabolism, along with issues in the expression of genes related to cellular stress responses, play an essential role in the disease (7, 8). Mitochondrial dysfunction is another major contributor (9–11). Mitochondria, organelles responsible for energy production, experience severe issues that impair ATP production, leading to neuronal energy deficits (10, 12). In Parkinson’s disease, complex I of the electron transport chain is particularly affected, resulting in increased oxidative stress and reactive oxygen species (ROS) production. Cellular oxidative stress arises when there is an imbalance between the overproduction of ROS and the body’s antioxidant defenses (13). Excessive ROS production, coupled with increased iron levels in the substantia nigra, promotes ROS formation, while antioxidant systems like glutathione are depleted (14, 15). This leads to oxidative damage to proteins, lipids, and DNA, ultimately causing cell death and α-synuclein aggregation (16–18).

Calcium homeostasis dysregulation severely affects neuronal function in Parkinson’s disease. Increased cytosolic calcium levels lead to excitotoxicity and neurodegeneration, as calcium overload activates cell death pathways (19). Abnormal calcium channel function increases the vulnerability of dopaminergic neurons, while disrupted calcium homeostasis impairs neurotransmitter release and synaptic plasticity (20, 21).

A hallmark of Parkinson’s disease is pathological protein aggregation, particularly the accumulation of misfolded α-synuclein (22). α-Synuclein aggregates form oligomers and fibrils, contributing to the formation of Lewy bodies (23–26). The aggregation of these abnormal proteins disrupts cellular functions, impairs protein clearance systems, and triggers neuroinflammation (18), and together with others (“phosphorylated tau and amyloid-beta”) “exacerbating neurodegeneration” (27). Furthermore, the prion-like spreading of misfolded α-synuclein proteins drives disease progression and interferes with axonal transport and synaptic function (28).

Impaired protein degradation pathways also play a crucial role in Parkinson’s disease. The ubiquitin-proteasome system dysfunction (UPS) results in the accumulation of damaged proteins (29–31). At the same time, the failure of the autophagy-lysosomal pathway leads to impaired lysosomal clearance of α-synuclein aggregates. Mutations in the PARK2 gene, which encodes parkin, further disturb the UPS, exacerbating the protein degradation issues (32).

Synaptic dysfunction contributes to both the motor and non-motor symptoms of Parkinson’s disease (33). The accumulation of α-synuclein impairs vesicle trafficking and neurotransmitter release, leading to altered dopamine release and uptake (34). Additionally, changes in synaptic plasticity affect other neurotransmitter systems, including cholinergic and serotonergic pathways, further contributing to the disease’s progression (35, 36).

Chronic neuroinflammation also plays a significant role in Parkinson’s disease progression (37, 38). Microglia and astrocytes are activated in response to cellular damage and protein aggregates, releasing pro-inflammatory cytokines such as TNF-α, IL-1β, and IL-6 (39, 40). Chronic neuroinflammation exacerbates oxidative stress and mitochondrial dysfunction, contributing to neuronal damage and increased blood-brain barrier permeability, which allows peripheral immune cells to infiltrate the central nervous system (41, 42).

Environmental factors further contribute to Parkinson’s disease development. Exposure to pesticides like Paraquat and Rotenone, organic solvents, metals such as iron and manganese, air pollution, viral infections, head trauma, and other stressors like PTSD can all play a role in the disease’s onset and progression (43, 44), and also alterations – including paraphysiological age-related – of the gut microbiota/gut microbiome, respectively in the functioning of the “microbiome-gut-brain axis, represented by both local and systemic effects of dysbiosis, including leaky gut with easier penetration of toxins through the intestinal barrier and multiple subsequent cascades mediated by bacterial metabolites” (45).

Parkinson’s disease is one of the most common neurological diseases in the elderly. It is the second most common neurodegenerative disease after Alzheimer’s disease (46).

The global incidence of PD is approximately 300,000 new cases recorded annually. PD affects 1%–3% of people over the age of 65, and almost 10 million people worldwide live with PD (46).

Parkinson’s disease has a male predominance, being 1.5 times more common in men than in women. In most cases, the onset of the disease occurs after the age of 55 (47).

Rarely, in 10%–15% of cases, the disease can onset at younger ages (48). In the United States, it is estimated that 4% of PD patients are diagnosed before the age of 50 (49).

With age, the incidence and prevalence of the condition increase. For instance, prevalence increases by 1% at age 60 and by 3% at age 80 (48, 50).

Almost a million Americans suffer from PD and this number is projected to increase to 1.2 million by 2030, since approximately 90,000 cases are diagnosed each year (49, 51).

As the global population ages, it is presumed that the number of those affected by PD will increase in Romania in the coming decades (52).

A relevant example of the “Evidence-based Medicine” (53) basic paradigm’s practical application is the use of quantitative (more or less structured) clinical (paraclinical)-functional assessment instruments, including in the – always – actual and most difficult domain of the neurax (brain and spinal cord) pathologies.

Specifically, but not exclusively, in PD rehabilitation, too, evaluation – as much as possible quantitative/standardized – periodically but sustained, is necessary for assessing the “entry level of the patient, to make a functional prognosis for recovery, to set rehabilitation goals, to evaluate the patient’s status over time and to guide therapy content and de-livery” (54).

It is to be emphasized an interesting and useful trend in this domain (55–58), i.e., to methodize frequently used clinical-functional assay scales/indices (obviously, a part of them available not only in PD), by framing/subsuming them within the actual World Health Organization (WHO)’s International Classification of Functioning, Disability and Health (ICF-DH) (59, 60) vision-structure/paradigm, on human functioning, according to their match with its components/domains: Body structures and, functions (B) and, respectively, Activities (A) and Participation (P), but also Contextual factors – including Environmental type “…barriers or facilitators to full participation for people with disabilities” (61).

**FIGURE 3:**
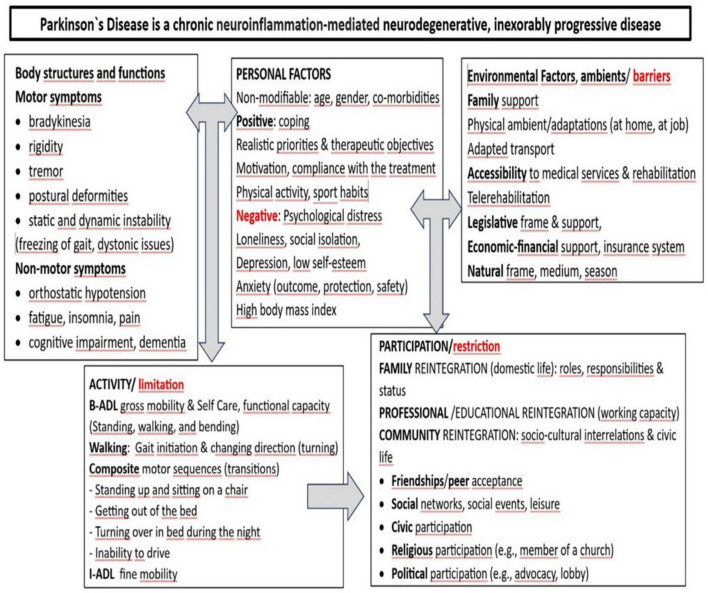
Synthetic presentation of the Parkinson’s disease (PD’) symptomatology framed/integrated within the International Classification of Functioning Disability and Health [ICF(-DH)] paradigm (66).

Therefore, within the general translational effort toward the International Classification of Functioning Disability and Health [ICF(-DH)] implementation, there is justified object to systematize and/or reappraisal periodically, including from this perspective, such clinical assessment instruments used to evaluate neuro-functional deficits, also in PD (see below a related synthesis, in Figure 3).

Large Language Models (LLMs) have received attention in healthcare, especially following the introduction of OpenAI’s Chat GPT in 2022 (62). LLM’s use in healthcare include a wide array of applications, including decision-making in clinical settings, diagnosis, analyzing diverse medical images and research, promising a solution for healthcare professionals to grapple with the ever-expending body of medical knowledge (63). This technology can potentially revolutionize the speed and efficiency of the way in which healthcare data is obtained, used and interpreted. Despite obvious benefits, it is mandatory to first evaluate LLM’s capacity to provide accurate and exhaustive data. Recent research highlighted the model’s tendency to supply “hallucinations,” providing incorrect, inaccurate information, false citations or references (64).

This paper outlines the capabilities of the current chat GPT model to provide accurate data for both research and diagnosis use in Parkinson’s disease. Finally, it summarizes the constraints and challenges of using LLM’s in this particular healthcare context, offering a new perspective of its shortcomings. In the medical field, where professionals’ primary focus is the patient’s outcomes, safety and wellbeing, incorrect, erroneous data can severely affect the patients’ ability to receive optimal care. Therefore, it is imperative to engage in regular reviews and updates of the LLMs to provide accurate data (65).

## 2 Methods

In our related knowledge base, acquired through human intelligence (HI), we used a balanced approach between documentation modalities: standardized, systematic literature review, and non-standardized, including works in the field freely discovered.

Consequently, we achieved a focused, step-by-step filtering/classification – according to the paradigm of the outspread literature identification and selection method: “Preferred Reporting Items for Systematic Reviews and Meta-Analyses (PRIS-MA)” (67) – initiated by the interrogation of some reputable international medical databases: Elsevier (68), The National Center for Biotechnology Information (NCBI)/PubMed (69), NCBI/PubMed Central (PMC) (69), Physiotherapy Evidence Database (PEDro) (70), and Institute for Scientific Information (ISI) Web of Knowledge/Science (71) (the latter for ISI indexing check of the journals where the identified articles have been published) using for the search specific combinations of keywords/syntaxes (see Table 1) contextually, over the period: 1*^st^* January 2018–31*^st^* December 2023.

**TABLE 1:** “The keyword combinations/syntaxes used contextually for this systematic literature review.

Keywords	Elsevier	PubMed	PMC	PEDro	Total
“Parkinson disease” AND (“assessment scales” + “nternational classification of functioning” + “body” + “activity” + “participation”) OR	14	0	6	0	20
“Parkinson disease” AND (“assessment scales” + “icf” + “body” + “activity” + “participation”) OR	21	0	5	0	26
“PD” + “assessment scales” AND (“international classification of functioning” + “body” + “activity” + “participation”) OR	7	0	7	0	14
“PD” AND (“assessment scales” + “icf” + “body” + “activity” + “participation”) OR	14	0	9	0	23
“Parkinson disease” AND (“assessment instruments” + “international classification of functioning” + “body” + “activity” + “participation”) OR	4	0	9	0	13
“Parkinson disease” AND (“assessment instruments” + “icf” + “body” + “activity” + “participation”) OR	5	0	10	0	15
“PD” AND (“assessment instruments” + “international classification of functioning” + “body” + “activity” + “participation”) OR	1	0	11	0	12
“PD” AND (“assessment instruments” + “icf” + “body” + “activity” + “participation”) OR	3	0	9	0	12
“Parkinson disease” AND (“assessment tools” + “international classification of functioning” + “body” + “activity” + “participation”) OR	25	0	26	0	51
“Parkinson disease” AND (“assessment tools” + “icf” + “body” + “activity” + “participation”) OR	30	0	27	0	57
“PD” + “assessment tools” AND (“international classification of functioning” + “body” + “activity” + “participation”) OR	12	0	30	0	42
“PD” AND (“assessment tools” + “icf” + “body” + “activity” + “participation”)	18	0	36	0	54
Total	154	0	185	0	339

**FIGURE 4:**
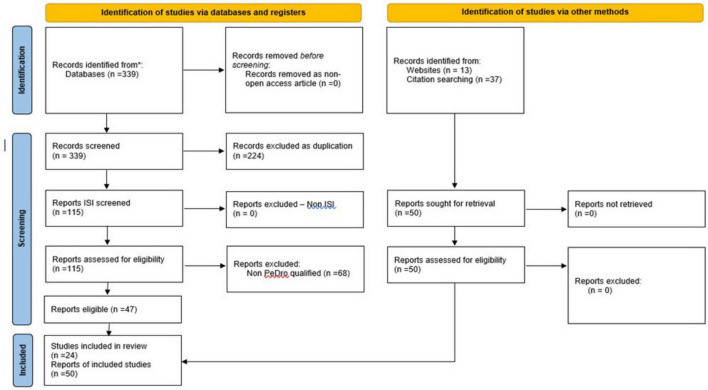
PRISMA type 2020 - flow diagram for new systematic reviews, which included searches of data-bases, registers and other sources applied to our standardized quest (67).

Regarding the paradigm underpinning our PEDro (72) inspired indirect quality classification of the selected articles, the basic criterion has been the weighted citations number per year, by a customized formula (73); finally, we have referred the respective average to the 0–10 points PEDro scale (72) (i.e., only 4–5 = fair quality, and higher – see Figure 4 and Table 2, for details) for each of the selected articles.

**TABLE 2:** The articles qualified/selected within our systematic literature review.

Article	Link	PEDro
Sevcenko, Ksenija; Lindgren, Ingrid. The effects of virtual reality training in stroke and Parkinson’s disease rehabilitation: a systematic review and a perspective on usability. *European Review of Aging and Physical Activity*, 2022, 19.1: 4. (74)	https://www.ncbi.nlm.nih.gov/pmc/articles/PMC8903585/	10
2. Ryan, Deirdre, et al. Effect of action observation therapy in the rehabilitation of neurologic and musculoskeletal conditions: a systematic review. Archives of rehabilitation research and clinical translation, 2021, 3.1: 100106. (75)	https://www.ncbi.nlm.nih.gov/pmc/articles/PMC7984987/	10
3. Di Lazzaro, Vincenzo, et al. Diagnostic contribution and therapeutic perspectives of transcranial magnetic stimulation in dementia. Clinical neurophysiology, 2021, 132.10: 2568–2607. (76)	https://www.sciencedirect.com/science/article/abs/pii/S1388245721006507?via%3Dihub	10
4. França, Carina, et al. Effects of cerebellar neuromodulation in movement disorders: a systematic review. *Brain stimulation*, 2018, 11.2: 249–260. (77)	https://www.researchgate.net/publication/321270618_Effects_of_cerebellar_neuromodulation_in_movement_disorders_A_systematic_review	10
5. Mackin, Roisin, et al. Neurophysiological markers of network dysfunction in neurodegenerative diseases. *NeuroImage: Clinical*, 2019, 22: 101706. (78)	https://www.ncbi.nlm.nih.gov/pmc/articles/PMC6370863/	10
6. Van Uem, Janet MT, et al. The association between objectively measured physical activity, depression, cognition, and health-related quality of life in Parkinson’s disease. *Parkinsonism & related disorders*, 2018, 48: 74–1. (79)	https://www.sciencedirect.com/science/article/pii/S1353802017308611?casa_token=3y8POG_SW9AAAAAA:KtomDCosTTiduQPSZL1weqbMUo_Rl_QrrFPRPq-e11Ltg7JCH_oOfIpRSvNySIMwqt3mC8Z7WxM	10
7. Bradnam, Lynley V., et al. Neurorehabilitation in dystonia: a holistic perspective. *Journal of Neural Transmission*, 2021, 128.4: 549–558. (80)	https://www.ncbi.nlm.nih.gov/pmc/articles/PMC8099801/	10
8. Schofield, Pat. The assessment of pain in older people: UK national guidelines. *Age and ageing*, 2018, 47.suppl_1: i1–i22. (81)	https://www.ncbi.nlm.nih.gov/pmc/articles/PMC5888957/	10
9. Barnish, Maxwell S.; Barran, Susannah M. A systematic review of active group-based dance, singing, music therapy and theatrical interventions for quality of life, functional communication, speech, motor function and cognitive status in people with Parkinson’s disease. *BMC neurology*, 2020, 20: 1–15. (82)	https://www.ncbi.nlm.nih.gov/pmc/articles/PMC7547481/	10
10. Bouça-Machado, Raquel; Maetzler, Walter; Ferreira, Joaquim J. What is functional mobility applied to Parkinson’s disease? *Journal of Parkinson’s disease*, 2018, 8.1: 121–130. (83)	https://www.ncbi.nlm.nih.gov/pmc/articles/PMC5836402/	10
11. Merchant, Kalpana M., et al. A proposed roadmap for Parkinson’s disease proof of concept clinical trials investigating compounds targeting alpha-synuclein. *Journal of Parkinson’s Disease*, 2019, 9.1: 31–61. (84)	https://www.ncbi.nlm.nih.gov/pmc/articles/PMC6398545/	10
12. Winser, Stanley J., et al. Measures of balance and falls risk prediction in people with Parkinson’s disease: a systematic review of psychometric properties. *Clinical rehabilitation*, 2019, 33.12: 1949–1962. (85)	https://www.ncbi.nlm.nih.gov/pmc/articles/PMC6826874/	10
13. Morya, Edgard, et al. Beyond the target area: an integrative view of tDCS-induced motor cortex modulation in patients and athletes. *Journal of neuroengineering and rehabilitation*, 2019, 16: 1–29. (86)	https://www.ncbi.nlm.nih.gov/pmc/articles/PMC6858746/	10
14. Joshi, Rajeshree, et al. PKG movement recording system use shows promise in routine clinical care of patients with Parkinson’s disease. *Frontiers in neurology*, 2019, 10: 1027. (87)	https://www.ncbi.nlm.nih.gov/pmc/articles/PMC6779790/	10
15. Stenum, Jan, et al. Applications of pose estimation in human health and performance across the lifespan. Sensors, 2021, 21.21: 7315. (88)	https://www.ncbi.nlm.nih.gov/pmc/articles/PMC8588262/	10
16. Etoom, Mohammad, et al. Therapeutic interventions for Pisa syndrome in idiopathic Parkinson’s disease. A Scoping Systematic Review. *Clinical Neurology and Neurosurgery*, 2020, 198: 106242. (89)	https://www.sciencedirect.com/science/article/abs/pii/S0303846720305850?via%3Dihub	8
17. Rumbach, Anna F.; Finch, Emma; Stevenson, Grace. What are the usual assessment practices in adult non-progressive dysarthria rehabilitation? A survey of Australian dysarthria practice patterns. Journal of communication disorders, 2019, 79: 46–57. (90)	https://www.sciencedirect.com/science/article/abs/pii/S0021992418300595?via%3Dihub	8
18. G̨iga, L., Pētersone, A., Čakstiòa, S. et al. Comparison of content and psychometric properties for assessment tools used for brain tumor patients: a scoping review. Health Qual Life Outcomes 19, 234 (2021). https://doi.org/10.1186/s12955-021-01863-0 (91)	https://www.ncbi.nlm.nih.gov/pmc/articles/PMC8501604/	7
19. Khedr, Eman M., et al. Are there differences in cortical excitability between akinetic-rigid and tremor-dominant subtypes of Parkinson’s disease? *Neurophysiologie Clinique*, 2021, 51.5: 443—53. (92)	https://www.sciencedirect.com/science/article/abs/pii/S0987705321000873?via%3Dihub	5
20. Seamon, Bryant A.; Kautz, Steven A.; Velozo, Craig A. Rasch analysis of the Activities-Specific Balance Confidence Scale in individuals poststroke. *Archives of rehabilitation research and clinical translation*, 2019, 1.3–4: 100028. (93)	https://www.ncbi.nlm.nih.gov/pmc/articles/PMC7170337/	5
21. Alghwiri, Alia A.; Almhdawi, Khader A.; Marchetti, Gregory. Are fatigue scales the same? A content comparison using the International Classification of Functioning, Disability and Health. *Multiple sclerosis and related disorders*, 2020, 46: 102596. (94)	https://www.sciencedirect.com/science/article/pii/S2211034820306702?casa_token=uohU0qQiKHMAAAAA:CmSRMtbV9DW_W29V4QcrSvQDWjT-2UYMF3D7feUHwhc-TwYB0xyN4fVnyu0AuReyjcAh1FpZHJQ	4
22. Heath, Gregory W.; Levine, David. Physical activity and public health among people with disabilities: research gaps and recommendations. *International Journal of Environmental Research and Public Health*, 2022, 19.16: 10436. (95)	https://www.ncbi.nlm.nih.gov/pmc/articles/PMC9408065/	4
23. Hsu, Tzu-Herng, et al. Large-scale assessment of function and disability in patients with parkinson’s disease using the functioning disability evaluation scale-adult version. *International journal of environmental research and public health*, 2018, 15.12: 2788. (96)	https://www.ncbi.nlm.nih.gov/pmc/articles/PMC6313551/	4
24. Raciti, Loredana, et al. Improving upper extremity bradykinesia in Parkinson’s disease: a randomized clinical trial on the use of gravity-supporting exoskeletons. Journal of Clinical Medicine, 2022, 11.9: 2543. (97)	https://www.ncbi.nlm.nih.gov/pmc/articles/PMC9104168/	4

We interrogated ChatGPT4.o and, respectively, ChatGPT Scholar, using the exact same keywords combinations/syntaxes, asking them to search contextually, meaning with same prompt content that was used in the HI PRISMA type search, hoping to acquire a satisfactory systematic literature review regarding assessment tools used in PD, that can adequately be compared to the HI generated version.

## 3 Results

As a contribution to the long and sustained efforts to systematize, from different perspectives, a growing number of assessment tools, including in PD, we have chosen to make it as simple as possible – as the more comprehensive and sophisticated are, the more time-consuming and therefore, more difficult to be applied in clinical practice will be, thus paradoxically with possibly inferior outcomes regarding their effective availability.

This aligns with excellent work done by Rissardo and Caprara (98), who searched for such assessment tools in three databases “until 2017.”

The authors made a very applied discussion upon their findings “114 scales … some …newly developed scales are incorporating part of other ratings instead of only citing them; this gives the medical literature a confusing assessment, when evaluating these grading systems, and turns them into long, confusing, and difficult to apply clinically” (98).

According to our aim, to make it simpler and therefore easier to be used in clinics, we further present, synthetically (tabular), the clinical-functional measurement tools found in our PRISMA type search, framed through the ICF domains and, respectively, also within an own related concept: (a) with (more) focused functional address and (b) with (more) global functional address.

Regarding the AI generated review, we started by interrogating ChatGPT4.o. Despite some progress, compared to ChatGPT3.5 – which asked if it can do a systematic literature review on the subject of our respective work (99) – answered: “As an AI language model, I do not have the capability to conduct a systematic review on Actovegin and ischemic stroke or any other research topic.”, ChatGPT4.o responded: “Yes, I can assist with creating a systematic literature review (SLR) following the PRISMA (Preferred Reporting Items for Systematic Reviews and Meta-Analyses) guidelines. PRISMA is a widely used methodology that ensures transparency, rigor, and reproducibility in systematic reviews.” ChatGPT Scholar responded similarly: “Certainly! I can guide you through the process of creating a Systematic Literature Review (SLR) using the PRISMA (Preferred Reporting Items for Systematic Reviews and Meta-Analyses) guidelines.”

As shown in Tables 3, 4, our research over the past 6 years has identified 60 frequently used assessment tools: 16 specifics to Parkinson’s and 44 covering various other conditions.

**TABLE 3:** Evaluation instruments with (more) focused functional address.

Evaluation instruments (scales/tests/questionnaires/index)	Acronym	ICF domain(s)	Used only in PD	Manifold used
1. Unified Dyskinesia Rating Scale (74–79, 82–85, 87–89, 92, 97)	UPDRS/UDysRS	B/A/P (100)	–	x
2. Purdue Pegboard Test (101)	PPBT	B (101) (A)*	–	x
3. Push and Release Test (85)	P&R	A (102)	–	x
4. Hoehn and Yahr Scale (74, 75, 79, 80, 83, 89, 92, 96, 97)	–	B/A/P*	x	–
5. Functional Gait Assessment (74, 85)	FGA	A (103)	–	x
6. Geriatric Depression Scale (74, 76, 79, 81)	GDS	B (104)	–	x
7. Numeric Pain Rating Scale (81, 97)	NPRS	B (105)	–	x
8. Dynamic Gait Index (74, 75, 85)	DGI	A (106) (B)*	–	x
9. 10 m walk test (74, 75)	10 MWT	A (107)	–	x
10. Four Square Step Test (85)	FSST	A (108)	–	x
11. Parkinson Psychosis Rating Scale (109)	PPRS	B/A/P*	x	–
12. Parkinson Psychosis Questionnaire (110)	PPQ	B/A/P*	x	–
13. Parkinson’s Disease Sleep Scale (111)	PPDS	B/A*	x	–
14. Sialorrhea Clinical Scale for PD (112)	SCS-PD	B*	x	–
15. Swallowing Disturbance Questionnaire (113)	SDQ	B/A*	–	x
16. Parkinson Neuropsychometric Dementia Assessment (114)	PANDA	B/A*	x	–

*Our adding/note.

**TABLE 4:** Evaluation instruments with (more) global functional address.

Evaluation instruments (scales/tests/questionnaires/index)	Acronym	ICF domain(s)	Used only in PD	Manifold used
1. Parkinson’s Disease Questionnaire-8 (115)	PDQ-8-	P (115) (A/B)*	x	–
2. Parkinson’s Disease Questionnaire-39 (74–76, 79, 82, 87, 89)	PDQ-39	P (A/B)*	x	–
3. Parkinson’s Disease Quality of Life Questionnaire (116)	PDQL	P*	x	–
4. Modified Parkinson Activity Scale (75)	M-PAS	B/A/P (117)	x	–
5. Profile PD (118)	Profile PD	B/A (118) (P)*	x	–
6. Parkinson Fatigue Scale (119, 120)	PFS -16	B/A/P (119)	x	–
7. Self-assessment Parkinson’s Disease Disability Scale (92)	SPDDS	A/P (121)	x	–
8. Parkinson’s Disease Activities of Daily Living Scale (79)	PADLS	A/P	x	–
9. Parkinson Impact Scale (122)	PIMS	A/P*	x	–
10. Australian Therapy Outcome Measures for Occupational Therapy (90)	AusTOMs-OT	B/A/P (123)	–	x
11. Freezing of Gait Questionnaire/New Freezing of Gait Questionnaire (75, 85, 86)	FOG-Q/NFOGQ	A (124)	x	–
12. Berg Balance Scale (74, 75, 77, 80, 82, 85, 89, 93)	BBS	A (125)	–	x
13. Timed “Up And Go” (74, 75, 77, 80, 82, 83, 85, 86, 88, 89, 93, 95)	TUG	B/A/P (126)	–	x
14. Borg Rating Scale of Perceived Exertion (74)	RPE	B (127)	–	x
15. Schwab and England Activities of Daily Living Scale (74–76, 81, 83, 86, 92)	SE-ADL/S&E	A (128)	–	x
16. Fatigue Severity Scale (74, 94)	FSS	A/P (120)	–	x
17. WHO Disability Assessment Scale (74, 81, 96)	WHODAS	A/P	–	x
18. 6 min Walk Test (74, 75, 89, 93)	6 MWT	A(129) (B)*	–	x
19. Nine-Hole Peg Test (77, 97)	9 HPT	B/A (130)	–	x
20. Tinetti Falls Efficacy Scale (80, 85)	Tinetti FES	A/P (131)	–	x
21. Tinetti Performance Oriented Mobility Assessment (75, 76, 82, 85, 91)	POMA; TMT	A (132)	–	x
22. EuroQol-5D (74, 81, 82, 89)	EQ-5D	B/P (133)	–	x
23. Voice-Related Quality of Life (82)	V-RQoL	B/A/P*	–	x
24. Communicative Participation Item Bank (82)	CPIB	P*	–	x
25. Functional Assessment of Chronic Illness Therapy – Fatigue Scale (94)	FACIT-Fatigue	B/A/P*	–	x
26. Sickness Impact Profile (81, 94)	SIP-68	B/A/P (134)	–	x
27. 36-Item Short Form Health Survey (74, 75, 81, 91, 93, 94)	SF-36	B/A/P*	–	x
28. Mini Mental State Examination (79, 82, 91)	MMSE	B (135)	–	x
29. Montreal Cognitive Assessment (82)	MoCA	B (136)	–	x
30. Frontal Assessment Battery (82, 137)	FAB	B/A*	–	x
31. Five Times Sit-to-Stand Test (83, 85)	FTSTS or 5xSTS	A (138)	–	x
32.Forward Functional Reach (85)	FFR	A (85)	–	x
33. Rapid assessment of postural instability questionnaire (85, 139)	RAPID	B/A/P*	x	–
34. Survey of Activities and Fear of Falling in the Elderly (140)	SAFE	A/P*	–	x
35. Total Energy Expenditure (79)	TEE	A (79)	–	x
36. WHO Quality of Life-BREF (98)	WHOQOL-BREF	A/P/Environment (141)	–	x
37. Activities-Specific Balance Confidence Scale (85)	ABC Scale	A (142)	–	x
38. Apathy Evaluation Scale (143)	AES	A/P (144)	–	x
39. Scales for Outcomes in PD-Autonomic (145)	SCOPA-AUT	B*	x	–
40. Non-Motor Symptoms Scale for Parkinson’s Disease (146)	NMSS	B*	x	–
41. Abnormal Involuntary Movement Scale (98)	AIMS	B/A*	–	x
42. Physical Anhedonia Scale (147)	PAS	B/A/P*	–	x
43. Short Parkinson’s Evaluation Scale/Scales for Outcomes in Parkinson’s disease (148)	SPES/SCOPA	B/A*	x	–
44. Scales for Outcomes in Parkinson’s Disease – Psychosocial Functioning (149)	SCOPA-PS	P*	x	–

*Our adding/note.

### 3.1 AI articles performed search

In parallel, we interrogated ChatGPT4.o and, respectively, ChatGPT Scholar. General information about the implementation of Chatbot AI models in various modern fields is concisely presented in one of our previous articles on Actovegin. By that time (2023), we availed ChatGPT3.5: released on 30 November 2022. As the next stage of development of ChatGPT3.5, the ChatGPT4 was released on 14 March 2023 (150).

With the continuously advancing AI evolution, ChatGPT4.o, was updated and presented as having larger processing and interaction facilities, with very large free accessibility, on May 2024 (151).

Among its capabilities: is the latest large multimodal language model that can accept and operate within multiple kinds of input (like images, videos, sounds, and texts) and generate content outputs on various professional and academic benchmarks (152).

The specialized Technology magazine emphasized that GPT4.o is 40% better than GPT3.5 on internal adversarial factuality evaluations, being capable of reducing fake information compared to previous bot models (153).

We have also directly interrogated it on its progress compared to GPT3.5, and herein are summarized all its related answers (pointing nine main directions):

“Improved Comprehension and Accuracy,” “Expanded Knowledge Base,” “Greater Contextual Awareness,” “Enhanced Problem-Solving Abilities,” “Reduced Biases and Ethical Improvements,” “Better Handling of Specific Domains,” “Improved Language Understanding Across Multiple Languages,” “Enhanced Creativity,” “Greater Reliability in Tool Integration.”

ChatGPT Scholar uses OpenAI’s ChatGPT and requires ChatGPT Plus to operate.

To assist scholars, the AI tool utilizes not only Google Scholar’s database but also other academic resources. It not only provides relevant data but also aids in comprehending and summarizing complex academic papers. Yet, it can access PubMed, but it is limited to 2022 (154).

We asked ChatGPT4.o and, respectively, ChatGPT Scholar to identify related works based on the same keywords’ combinations/syntaxes, searched contextually, i.e., with the same interrogation contents as we did for our related PRISMA type HI achieved quest to fulfill a systematic literature review regarding assessment tools used in PD.

In our dialog with ChatGPT4.o and, respectively, ChatGPT Scholar, we informed them that we want they interrogate for a PRISMA type systematic literature review the following well-known recognized internationally databases: Elsevier (NCBI)/PubMed, NCBI/(PMC), PEDro (as we have an already long and successful experience in achieving PRISMA type systematic literature reviews through interrogating these refutable medical databases) (3, 99, 155–169).

To this request of ours, Chat GPT4.o gave us the following answer: “I cannot access external databases like Elsevier, NCBI/PubMed, PubMed Central (PMC), or PEDro directly within this environment. However, I can help guide you on how to perform these searches yourself on the respective platforms” (see Supplementary Annex 1).

ChatGPT Scholar did not declined its availability to address the medical data-bases. Its answer was: “Could you please provide the specific word combinations or syntaxes you would like me to use for the search? This will help me conduct a more accurate and tailored search based on your requirements” (see Supplementary Annex 3) but the results provided were inappropriate (see Supplementary Annex 4).

We have asked them every of the 12 above presented keywords combinations/syntaxes, to be addressed contextually, each of it twice and every time from different computers (IPs).

We have received from ChatGPT4.o and ChatGPT Scholar different results of their search, not acceptably/compatible neither with our HI-made PRISMA type systematic literature review nor with their findings in between the two interrogations realized contextually, for all the 12 keywords combinations/syntaxes; and this created a con-fusing situation (see Supplementary Annexes 2, 4).

### 3.2 Funnel plot analysis

**FIGURE 5:**
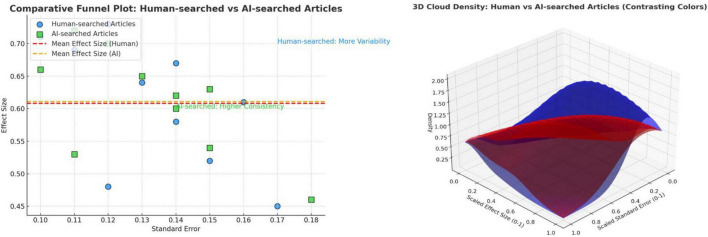
**(a)** The funnel plot for human intelligence (HI)-searched vs. artificial intelligence (AI)-searched articles; **(b)** Visual density representation.

The analysis aims to explore potential publication bias in both datasets and pro-vide insights into the risk of bias in between the two type of approaches: HI vs. AI driven related searches (Figure 5). Data were extracted from the 24 human-searched articles and the corresponding set of AI-searched works. Key metrics such as effect size, standard error, and sample size were obtained from the studies using estimation functions. The funnel plot was generated to visually compare the distribution of effect sizes and standard errors for both datasets. The degree of symmetry and clustering in the funnel plot was used to assess the likelihood of publication bias.

According to our Funnel plot analysis, HI appears to outperform AI in systematic literature searches, especially in finding articles of consistently higher quality, yet the risk of bias still exists. While AI can retrieve relevant results, it may introduce greater variability, leading to the inclusion of lower-impact studies. This suggests that while AI tools are improving, they still lag behind human expertise in certain specialized tasks like systematic literature reviews. However, AI-driven endeavors might have some capabilities which, because of their machine-automated functioning paradigm, could lead to progressively improving mitigation of the bias risk (see details in Supplementary Annex 5).

Considering the complexity of both: PD and the arising AI-driven tools era, we considered appropriate to extend the area of our systematic literature review with – but because of the tremendously fast developing of the latter – just a narrative one, especially dedicated to the contribution of such AI facilities at the accuracy improvement of the neurofunctional assessments within biomarker-based frameworks.

Accordingly, we have interrogated (again) ChatGPT4.o and ChatGPT Scholar and herein bellow there is a synthesis of the results showing their recently related capabilities.

Related interrogation of ChatGPT4.o:

“1. Neuroimaging analysis

AI techniques, particularly deep learning, transform neuroimaging analysis by uncovering subtle structural and functional changes in the brain associated with Parkinson’s disease. Related key contributions include:

• Structural Imaging (MRI): AI algorithms analyze structural MRI scans to detect thinning in specific brain regions such as the substantia nigra, which is a hallmark of PD. Advanced image segmentation techniques highlight subtle atrophy or abnormalities in gray and white matter that are often missed by human observation.
• Functional Imaging (fMRI, PET): Functional MRI and PET scans provide data on brain activity and dopa-mine metabolism. AI tools identify abnormal patterns in brain connectivity, such as disruptions in motor and cognitive networks. PET imaging combined with AI can also measure dopamine transporter (DAT) binding, improving early diagnosis.
• Diffusion Tensor Imaging (DTI): AI enhances the assessment of microstructural changes in white matter tracts using DTI. These models detect changes in fractional anisotropy and mean diffusivity, linked to neurodegeneration.
• Biomarker Discovery: AI aids in discovering imaging biomarkers by automatically analyzing datasets to predict disease progression or differentiate PD from similar disorders like multiple system atrophy (MSA) or progressive supranuclear palsy (PSP).
  2. Integration of multimodal biomarkers

Parkinson’s disease diagnosis and progression involve multiple biomarkers from diverse sources. AI excels at integrating these to build more robust and comprehensive models. Specific advancements include:

• Genetic and Proteomic Data: Genetic mutations such as LRRK2, GBA, or SNCA and protein biomarkers like alpha-synuclein and tau in cerebrospinal fluid (CSF) are critical for PD. AI platforms combine these genetic and biochemical data with clinical phenotypes to classify disease subtypes or predict progression rates.
• Multimodal Fusion: Combining imaging, electrophysiological, and molecular biomarkers is complex. AI models, such as multi-task learning, integrate heterogeneous datasets to capture disease patterns comprehensively, enabling the creation of personalized profiles for patients.
• Differential Diagnosis: By integrating diverse biomarkers, AI improves the differentiation of Parkinson’s disease from other neurodegenerative disorders. For example, machine learning models can distinguish between PD, essential tremor, and atypical parkinsonism by analyzing biomarker profiles.

3. Wearable and sensor data processing

• Wearable technologies, such as smartwatches, accelerometers, and pressure sensors, generate continuous data streams related to motor and non-motor symptoms. AI-driven tools analyze this data to provide objective, real-time assessments:
• Motor Symptom Quantification: AI analyzes wearable sensor data to quantify tremors, bradykinesia (slowness of movement), rigidity, and gait abnormalities. Algorithms can evaluate patterns in movement speed, stride length, and balance, offering accurate symptom scoring aligned with clinical scales like the Unified Parkinson’s Disease Rating Scale (UPDRS).
• Fluctuation Detection: Parkinson’s symptoms often fluctuate throughout the day. AI-powered wearables detect and record these fluctuations, enabling clinicians to fine-tune medications (e.g., levodopa dosing) to improve symptom control.
• Remote Monitoring: Wearable devices connected to AI systems allow patients to be monitored remotely, reducing the need for frequent clinic visits. These tools are particularly valuable in tracking long-term dis-ease progression.
• Fall Detection: Falls are a major risk for PD patients. AI embedded in wearable sensors can predict and detect falls, providing early warnings or alerts to caregivers.

4. Prediction and Monitoring of Disease Progression

• AI models analyze large datasets from patients to predict the trajectory of Parkinson’s disease and optimize treatment strategies:
• Disease Progression Prediction: Machine learning algorithms analyze longitudinal data (e.g., clinical exams, imaging, and biomarkers) to predict the rate of disease progression. For example, they can estimate when a patient may transition from mild to advanced stages or develop complications like dyskinesia.
• Therapeutic Response Analysis: AI helps monitor the efficacy of treatments such as dopaminergic therapies, deep brain stimulation (DBS), or experimental drugs. By analyzing symptom changes over time, these tools help clinicians adjust therapies to maximize benefits and minimize side effects.
• Risk Stratification: AI identifies patients at higher risk of rapid progression or specific complications, enabling earlier interventions or inclusion in tailored clinical trials.

5. Cognitive and Behavioral Assessment

• Non-motor symptoms such as cognitive decline, depression, and speech impairments significantly impact PD patients. AI-driven tools expand the scope of neurofunctional assessments by evaluating these complex domains:
• Cognitive Decline: AI algorithms analyze neuropsychological test results, imaging, and biomarker data to detect early cognitive impairment. This is critical, as mild cognitive impairment (MCI) in PD can progress to dementia in some patients.
• Speech and Voice Analysis: AI-based natural language processing (NLP) and acoustic analysis tools assess speech and voice features. For example, they can measure reduced speech volume, monotonicity, and articulation difficulties associated with PD. Such tools are sensitive enough to detect early speech changes that might not be noticeable in clinical settings.
• Emotion and Behavior Monitoring: AI-powered sentiment analysis and facial recognition algorithms assess emotional states such as depression or apathy, which are common in PD. These tools provide additional insights into the overall functional status of patients.
• Digital Behavioral Biomarkers: AI uses data from digital apps or sensors to track behavioral changes over time, such as reduced activity levels, changes in typing speed, or altered daily routines, providing indirect measures of disease impact.

AI-driven tools are revolutionizing neurofunctional assessments in Parkinson’s disease by improving the precision, integration, and scope of biomarker analysis. These advancements enable earlier diagnosis, more accurate monitoring of disease progression, and personalized therapeutic approaches. By combining insights from neuroimaging, wearable sensors, multimodal biomarkers, and behavioral data, AI creates a holistic framework for managing Parkinson’s disease, improving patient outcomes, and advancing research.”

After asking ChatGPT for an algorithm to help diagnose in the early stages of Parkinson’s disease, here is what it replied:

“Diagnosis Recommendation:

- Early Diagnosis: If two or more criteria are met, including at least one biomarker abnormality.
- Probable PD: If 3+ categories show abnormalities, including neuroimaging or genetic markers.
- Definitive Diagnosis: Confirm with biomarker evidence (e.g., DAT scans) and progression tracking.”

Related interrogation of ChatGPT Scholar:

“The integration of artificial intelligence (AI) with multi-omics data (genomics, epigenomics, proteomics, transcriptomics, metabolomics, and other biological layers) has opened new opportunities for understanding and managing Parkinson’s disease (PD). This approach enables the discovery of new biomarkers, the understanding of pathological mechanisms, and the development of personalized treatments.

**Table:** 

Category	Feature	Criteria for diagnosis
Motor symptoms	Tremor, Bradykinesia, Rigidity, Gait Abnormality	Presence of 2 + motor symptoms based on clinical scales (e.g., UPDRS)
Neuroimaging biomarkers	DAT Binding Abnormalities (PET), Structural Atrophy (MRI)	Reduced DAT binding or atrophy in substantia nigra region
Wearable sensor data	Movement Patterns, Gait, Tremor Analysis	Abnormal gait speed, tremor frequency, or rigidity detected via AI-based wearable analysis
Genetic markers	LRRK2, GBA, SNCA Mutations	Presence of known PD-associated genetic mutations
Cognitive and behavioral	Speech Impairments, Cognitive Decline	Early speech or cognitive decline detected through AI-based language and neuro-psych tests
Risk Factors	Age, Family History, Environmental Exposures	Higher risk factors (age > 60, family history, pesticide exposure) combined with symptoms

#### 3.2.1 The role of multi-omics data in Parkinson’s disease

Parkinson’s disease is a complex neurodegenerative disorder characterized by the progressive loss of dopaminergic neurons and the accumulation of alpha-synuclein protein. Multi-omics data offer diverse insights:

Genomics: Studies genetic variations (e.g., mutations in LRRK2, SNCA, PARK7) that predispose individuals to PD.

Epigenomics: Investigates epigenetic changes (DNA methylation, histone modifications) regulating gene expression without altering DNA sequences.

Proteomics: Analyzes proteins involved in PD, such as alpha-synuclein and their post-translational modifications.

Transcriptomics: Examines changes in messenger RNA and microRNAs that influence neuronal functions.

Metabolomics and lipidomics: Identifies metabolic dysfunctions and lipid alterations in the brains of PD patients.

#### 3.2.2 The role of AI in multi-omics analysis

AI plays a central role in processing and analyzing the large volumes of multi-omics data using techniques such as machine learning (ML) and deep learning (DL). Key applications include:

Biomarker discovery: AI algorithms identify unique molecular signatures that differentiate PD patients from healthy individuals. For instance, AI can combine genomic and proteomic data to detect novel diagnostic biomarkers.

Integrative analysis: AI models integrate multi-omics data to uncover complex biological networks contributing to PD, such as gene-protein interactions.

Disease progression prediction: Predictive models based on AI can evaluate PD severity and progression using biomarkers from multiple omics layers.

Personalized medicine: Integrating patient-specific data with AI models enables the design of tailored treatments based on individual molecular profiles.

#### 3.2.3 Practical applications

##### Biomarker identification

An integrative multi-omics study using AI identified a group of specifically altered proteins in PD patients by analyzing proteomic and transcriptomic data. These biomarkers can be used for early diagnosis or therapy monitoring.

##### Understanding disease mechanisms

Artificial intelligence has been applied to analyze epigenomic and transcriptomic data, identifying changes in genes involved in neuronal inflammation and mitochondrial functions. These findings provide new perspectives on neuroprotective therapies.

##### Predicting treatment response

Machine learning models have been used to analyze genedrug interactions and predict patient responses to dopaminergic treatments based on genetic profiles.

#### 3.2.4 Challenges and future perspectives

##### Challenges

Data heterogeneity: Integrating multiple types of omics data requires robust harmonization methods.

Large data volumes: Requires high-performance computational infrastructure for storage and processing.

Reproducibility: Results across studies can vary, necessitating standardization.

##### Future perspectives

Advanced algorithms: Developing AI algorithms capable of learning complex relationships between multi-omics data and PD phenotypes.

Integrated platforms: Creating platforms to collect and analyze multi-omics data for PD diagnosis and treatment.

Liquid biopsies: Using AI to analyze multi-omics data from biological fluids (plasma, CSF) to detect minimally invasive biomarkers.

The integration of AI with multi-omics data is essential for advancing the understanding and treatment of Parkinson’s disease. This approach enables better patient stratification, the discovery of new therapeutic targets, and the personalization of treatments, offering hope for more effective management of the disease.”

The information provided by ChatGPT4.o and ChatGPT Scholar is derived from multiple articles they quoted [check (170–178)].

## 4 Discussion

With the rapid advancements in large language models like ChatGPT, the academic community has been considering the potential impact on related integrity and research practice.

In the rapidly expanding field of AI, various other AI models, facilities, and tools have emerged and continue to appear, such as Perplexity AI (179, 180) and JenniAI, respectively. Interestingly when asking JenniAI about ChatGPT’s capabilities in demonstrating complex reasoning and delivering coherent responses to university-level questions, it also emphasized the strengths and limitations of AI tools, specifically ChatGPT, in the context of education and research. Unlike search engines, which can provide direct links to source materials, ChatGPT does not include URLs or other metadata, making it difficult for users to verify information sources. It raises concerns about the correctness and integrity of the research process.

Further analysis of ChatGPT’s capabilities in this area has revealed issues in understanding academic concepts and accurately citing sources or providing complete bibliographic information (181).

Being trained on data selected from various sources, it has a certain level of bias, reflecting these bibliographic sources.

The ChatGPT4.o version found 1 article and, respectively, ChatGPT Scholar found two references, all fulfilling our search pattern by afford mentioned syntaxes, but out of the period we have searched our PRISMA type systematic literature review (published in 2024, whereas our search period 1^st^ January 2018–31^st^ December 2023) and the second was not access free (see Supplementary Annexes 2, 4).

Responsible use of ChatGPT involves being aware of these limitations and verifying the information to ensure the accuracy and relevance of results. The AI model offers recommendations for the direct search of academic databases, indicating web-sites of consecrated platforms.

OpenAI is preparing to launch a new, stronger version of ChatGPT: ChatGPT-5 that will be a “significant leap” in AI (182).

## 5 Conclusion

Considering the answers we have received from the two above interrogated AI models (and presented in the Supplementary Annexes 2, 4 – contained in the related Supplementary materials), we reckon they still cannot achieve systematic literature reviews at rigor standards – at specially regarding high quality identified papers – comparable to those fulfilled by HI PRISMA.

On the other hand, we have determined that such AI-driven tools became lately able to improve the intimate evaluation dimension represented by biomarkers availing also advanced related diagnostic algorithms.

But, hopefully their capability’s progress will continue, obviously with the great attention the development of AI must be conducted and surveyed – see, in this respect, the recent related European Union (EU) related regulation (183) – thus avoiding “the risk of a Sorcerer’s Apprentice kind of behavior” (99) in this very important contemporary topic.

Our research is not intended to “crush the miracle corolla of the world“ – as a well-known Romanian poet says (184) –but to strengthen the confidence, at least for now, in the classic HI academic way of caring out Systematic Literature Reviews, following the large recognized, standardized PRISMA type, methodology.

## Data Availability

The original contributions presented in this study are included in this article/Supplementary material, further inquiries can be directed to the corresponding authors.
