# Correction: Standardized clinical assessments and advanced AI-driven instruments used to evaluate neurofunctional deficits, including within biomarker based framework, in Parkinson's disease - human intelligence made vs. AI models - systematic review

**DOI:** 10.3389/fmed.2025.1649641

**Published:** 2025-07-14

**Authors:** Aurelian Anghelescu, Constantin Munteanu, Aura Spinu, Vlad Ciobanu, Cristina Popescu, Ioana Elena Cioca, Ioana Andone, Simona-Isabelle Stoica, Mihaela Mandu, Ana Rebedea, Sebastian Giuvara, Alin-Daniel Malaelea, Andreea-Iulia Vladulescu-Trandafir, Maria-Veronica Morcov, Gelu Onose

**Affiliations:** ^1^Department of Specific Discipline, Faculty of Midwifery and Nursing, University of Medicine and Pharmacy “Carol Davila”, Bucharest, Romania; ^2^Teaching Emergency Hospital “Bagdasar-Arseni”, Bucharest, Romania; ^3^Department of Biomedical Sciences, Faculty of Medical Bioengineering, University of Medicine and Pharmacy “Grigore T. Popa”, Iaşi, Romania; ^4^Department of Clinical Education, Physical and Rehabilitation Medicine, Faculty of Medicine, University of Medicine and Pharmacy “Carol Davila”, Bucharest, Romania; ^5^Department of Computer Science, Politehnica University of Bucharest, Bucharest, Romania; ^6^Department of Basic Medical Sciences, Faculty of Midwifery and Nursing, University of Medicine and Pharmacy “Carol Davila”, Bucharest, Romania; ^7^National Teaching Centre for Neuro-psycho-motor Rehabilitation in Children “Dr. N. Robanescu”, Bucharest, Romania

**Keywords:** Parkinson's disease, human intelligence, artificial intelligence, assessment instruments, ICF framing, ChatGPT4.o, ChatGPT Scholar

In the published article, there was an error in the affiliations for the following authors:

Aura Spinu, Cristina Popescu, Ioana Andone, Mihaela Mandu, Andreea-Iulia Vladulescu-Trandafir, and Gelu Onose.

These authors were originally listed with the affiliations:

“Teaching Emergency Hospital ‘Bagdasar-Arseni', Bucharest, Romania” and “Department of Clinical Education, Physical and Rehabilitation Medicine, Faculty of Medicine, University of Medicine and Pharmacy ‘Carol Davila', Bucharest, Romania”.

The affiliation for Aura Spinu, Cristina Popescu, Ioana Andone, Mihaela Mandu, Andreea-Iulia Vladulescu-Trandafir, and Gelu Onose should only be:

“Department of Clinical Education, Physical and Rehabilitation Medicine, Faculty of Medicine, University of Medicine and Pharmacy “Carol Davila”, Bucharest, Romania.”

This correction does not affect the scientific content, results, or conclusions of the article in any way. The original article has been updated accordingly.

