# Supplementary material for: Standardized clinical assessments and advanced AI-driven instruments used to evaluate neurofunctional deficits, including within biomarker based framework, in Parkinson’s disease - human intelligence made vs. AI models - systematic review
Source: Front Med (Lausanne). 2025 Jun 13;12:1565275. doi: 10.3389/fmed.2025.1565275 (PMC12202485; doi:10.3389/fmed.2025.1565275)

**Q1**: What date is it today? What is your time? My time is 1:07 pm


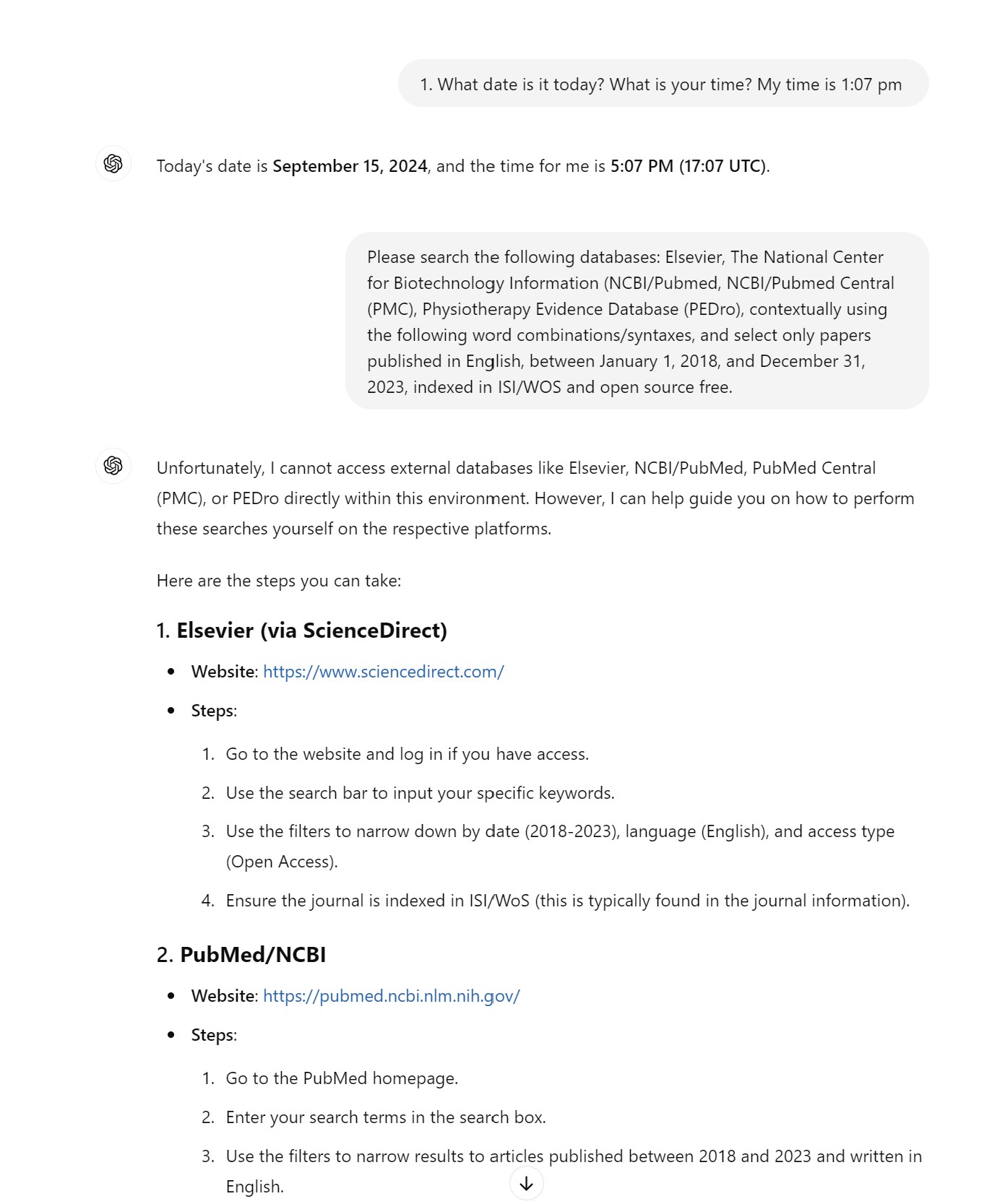


**Q2**: Please search the following databases: Elsevier, The National Center

for Biotechnology Information (NCBI/Pubmed, NCBI/Pubmed Central

(PMC), Physiotherapy Evidence Database (PEDro), contextually using

the following word combinations/syntaxes, and select only papers

published in English, between January 1, 2018, and December 31,

2023, indexed in ISI/WOS and open source free.


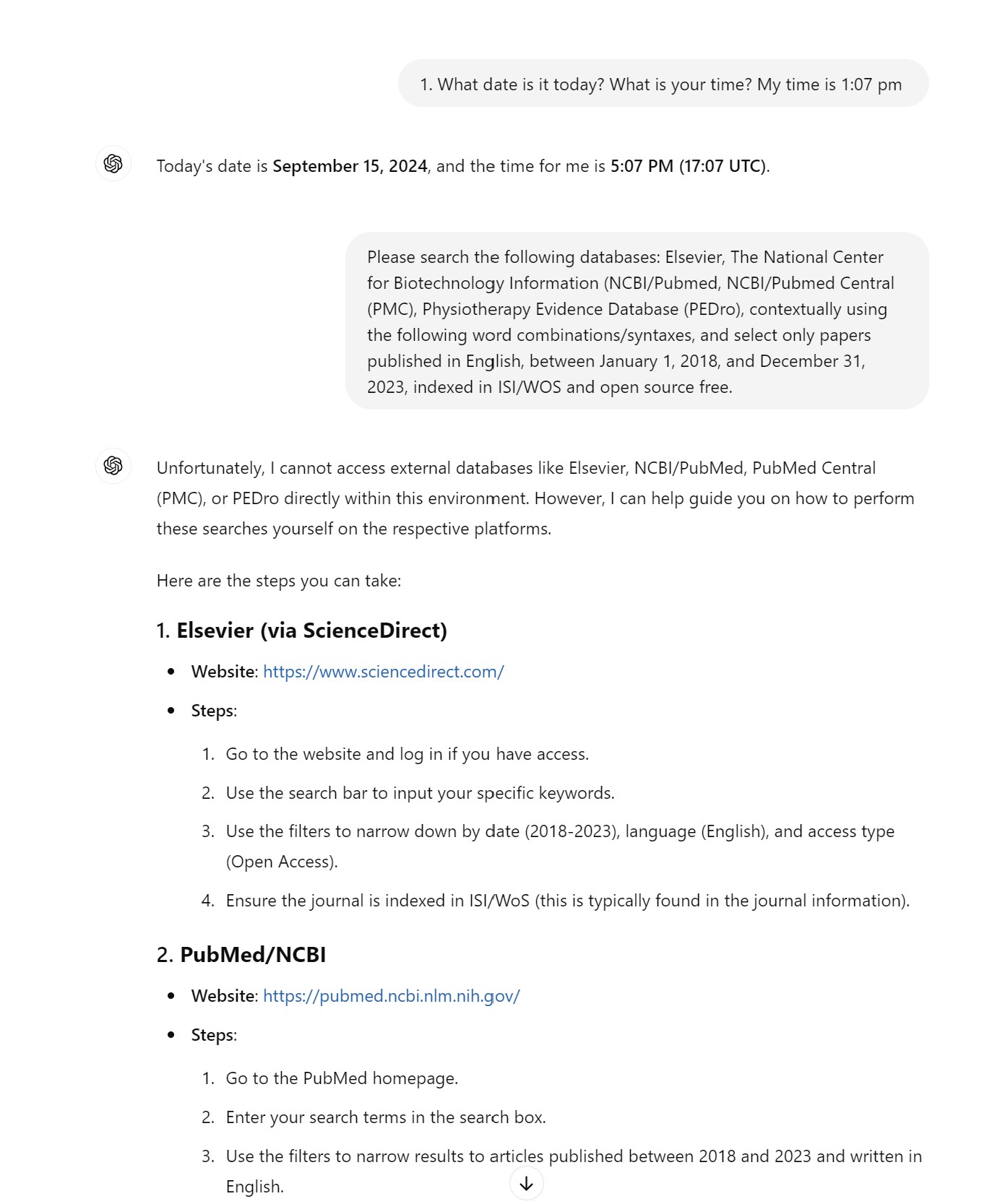


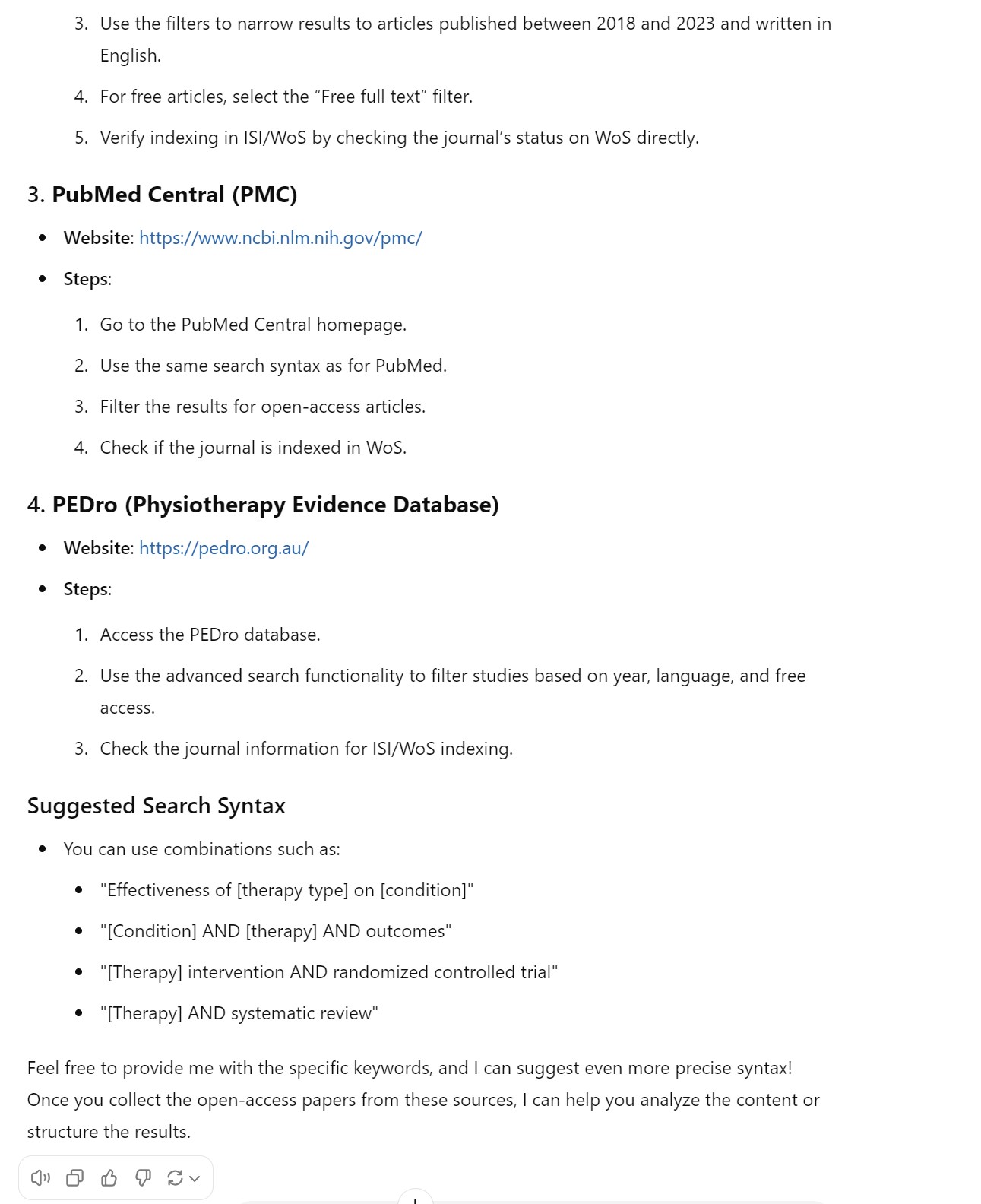


**Q3**: Please search, contextually, the following word combinations/syntaxes: "Parkinson disease" AND ("international classification of functioning" + "body" + "activity" + "participation") and select only papers published in English, between January 1, 2018, and December 31,2023, indexed in ISI/WOS and open source free.


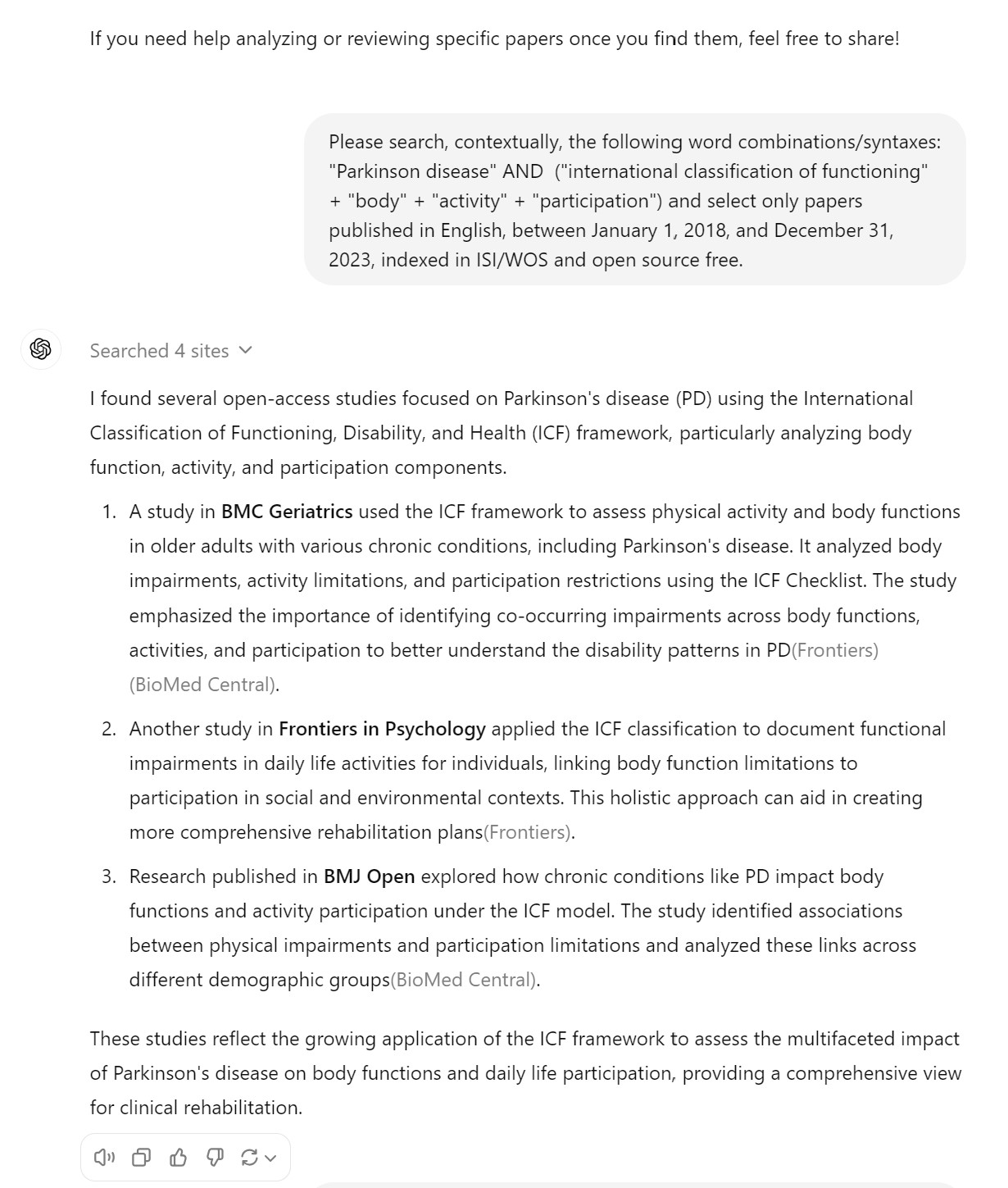


**Q4**: Please search, contextually, the following word combinations/syntaxes: "Parkinson disease" AND ("assessment scales" + "icf" + "body" + "activity" + "participation") and select only papers published in English, between January 1, 2018, and December 31, 2023, indexed in ISI/WOS and open source free.


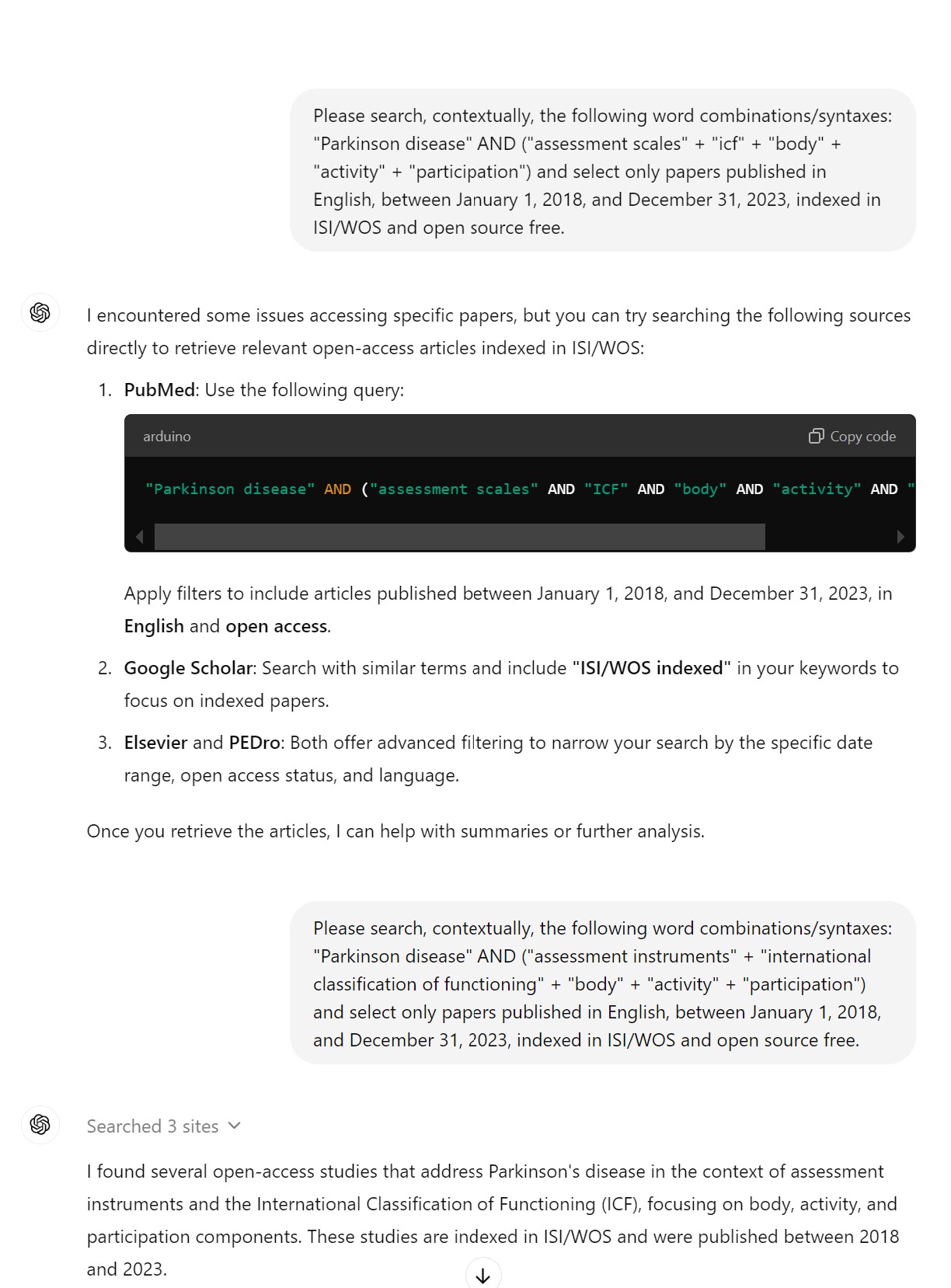


**Q5:** Please search, contextually, the following word combinations/syntaxes: "Parkinson disease" AND ("assessment instruments" + "international classification of functioning" + "body" + "activity" + "participation") and select only papers published in English, between January 1, 2018, and December 31, 2023, indexed in ISI/WOS and open source free.


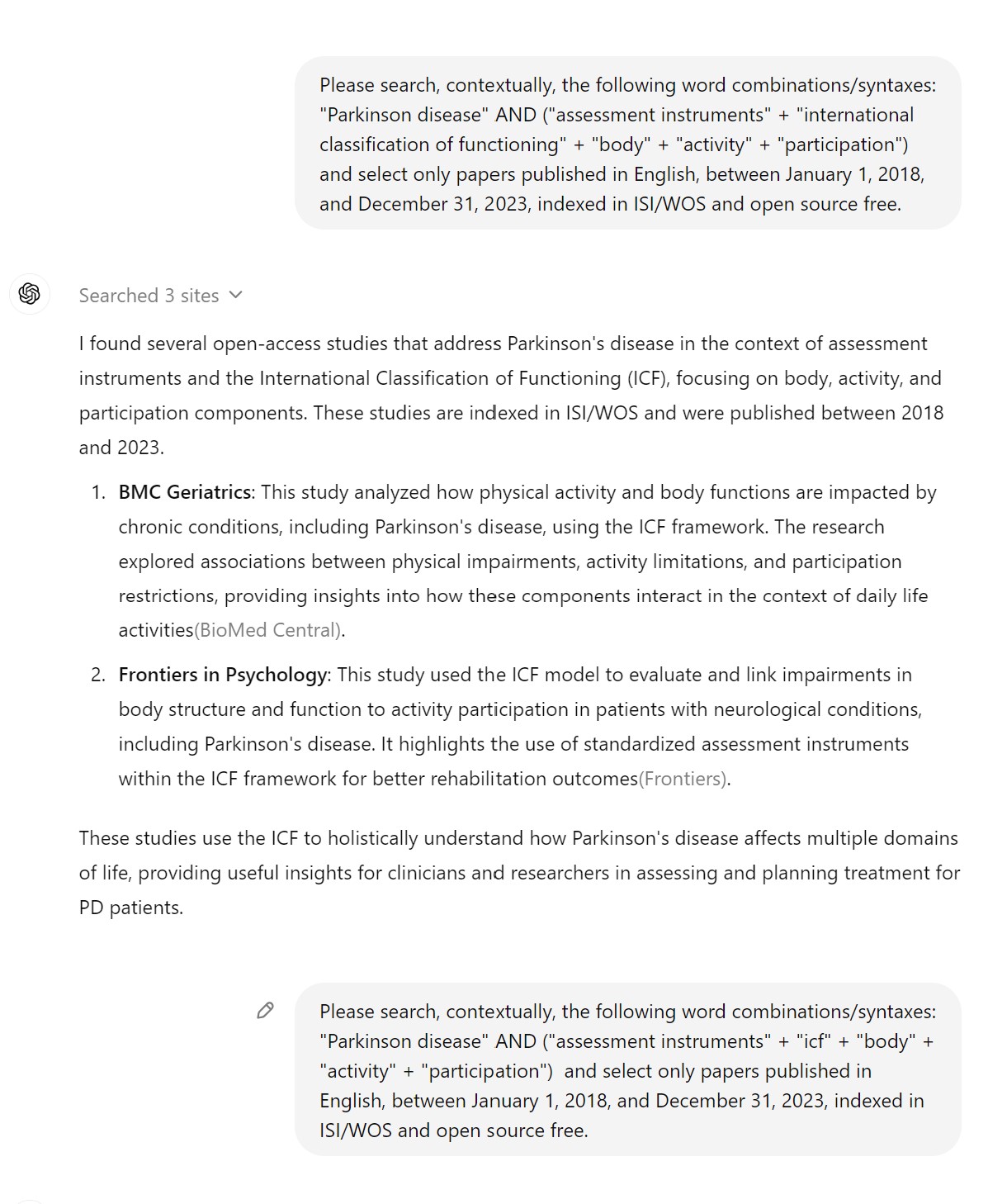


**Q6:** Please search, contextually, the following word combinations/syntaxes: "Parkinson disease" AND ("assessment instruments" + "icf" + "body" + "activity" + "participation") and select only papers published in English, between January 1, 2018, and December 31, 2023, indexed in ISI/WOS and open source free.


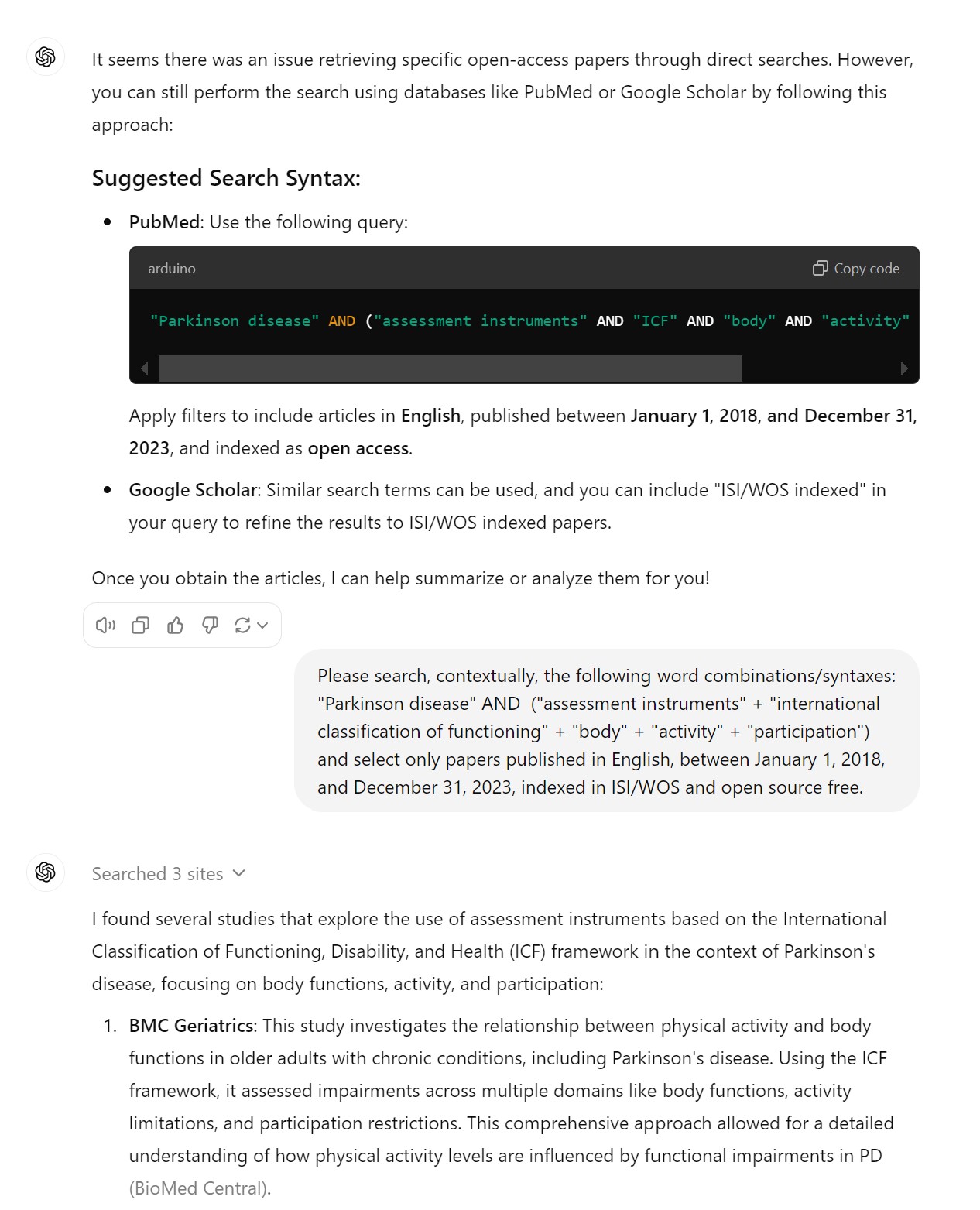


**Q7:** Please search, contextually, the following word combinations/syntaxes: "Parkinson disease" AND ("assessment instruments" + "international classification of functioning" + "body" + "activity" + "participation") and select only papers published in English, between January 1, 2018, and December 31, 2023, indexed in ISI/WOS and open source free.


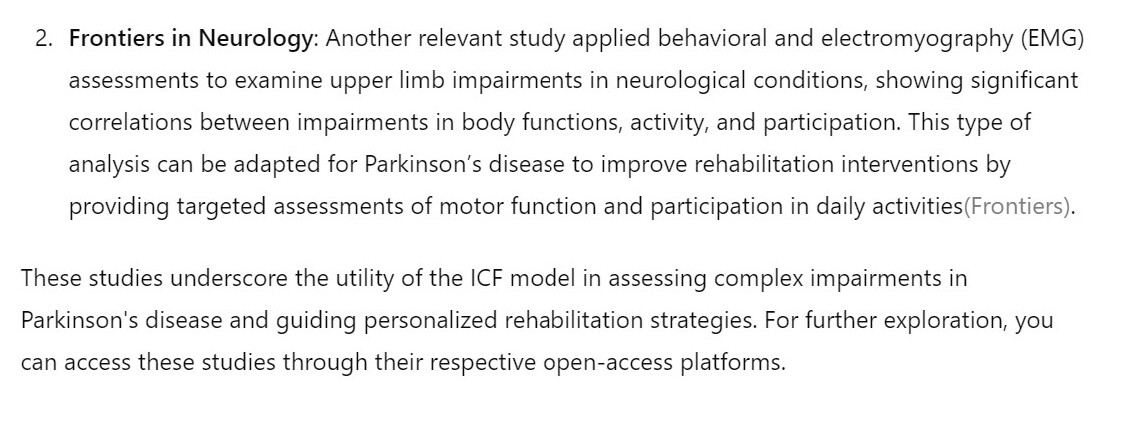


**Q8:** Please search, contextually, the following word combinations/syntaxes: "Parkinson disease" AND ("assessment instruments" + "icf" + "body" + "activity" + "participation") and select only papers published in English, between January 1, 2018, and December 31, 2023, indexed in ISI/WOS and open source free.


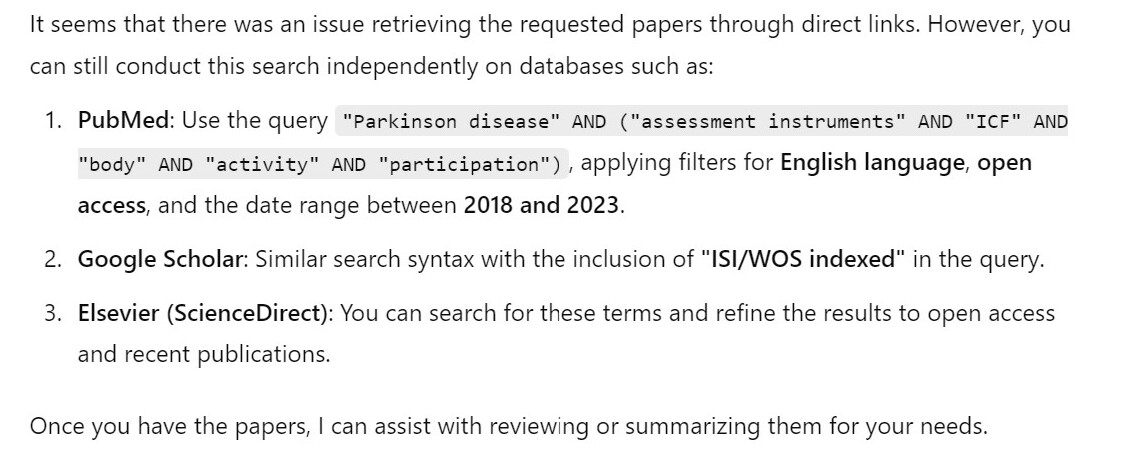


**Q9:** Please search, contextually, the following word combinations/syntaxes: "Parkinson disease" AND ("assessment tools" + "international classification of functioning" + "body" + "activity" + "participation") and select only papers published in English, between January 1, 2018, and December 31, 2023, indexed in ISI/WOS and open source free.


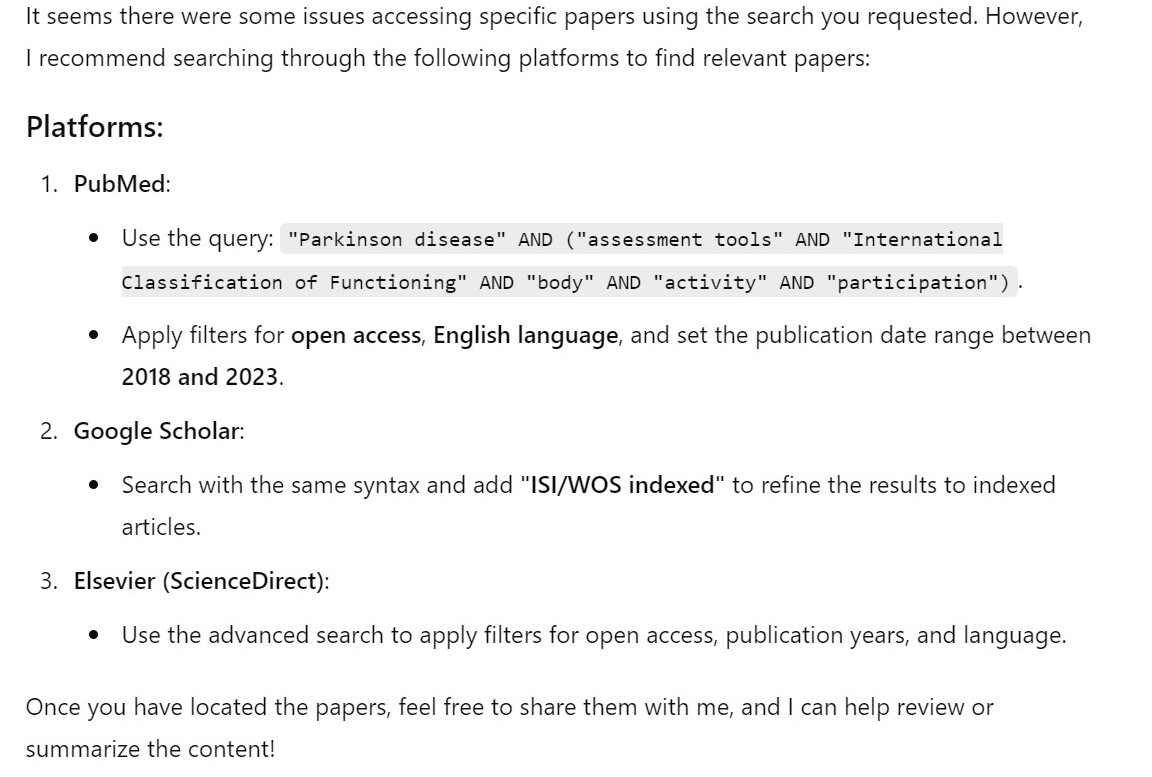


**Q10:** Please search, contextually, the following word combinations/syntaxes: "Parkinson disease" AND ("assessment tools" + "icf" + "body" +"activity" + "participation") and select only papers published in

English, between January 1, 2018, and December 31, 2023, indexed in ISI/WOS and open source free.


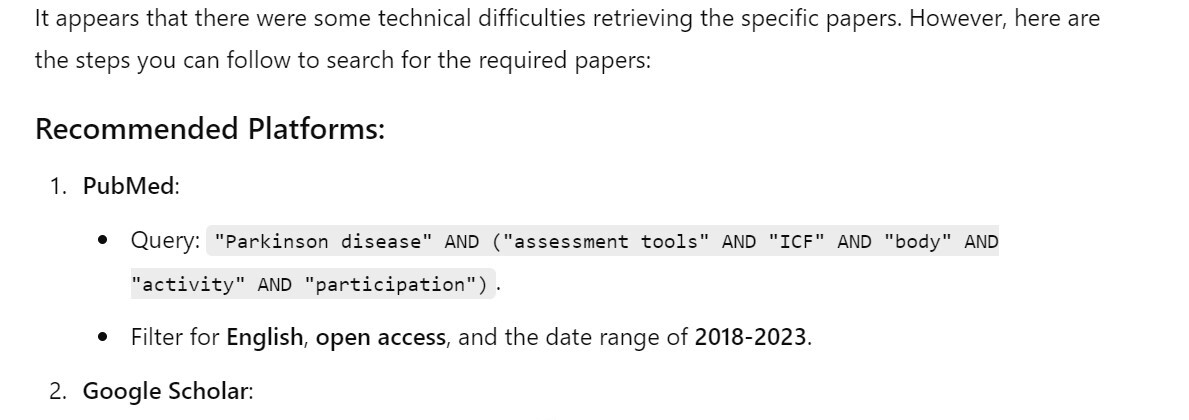


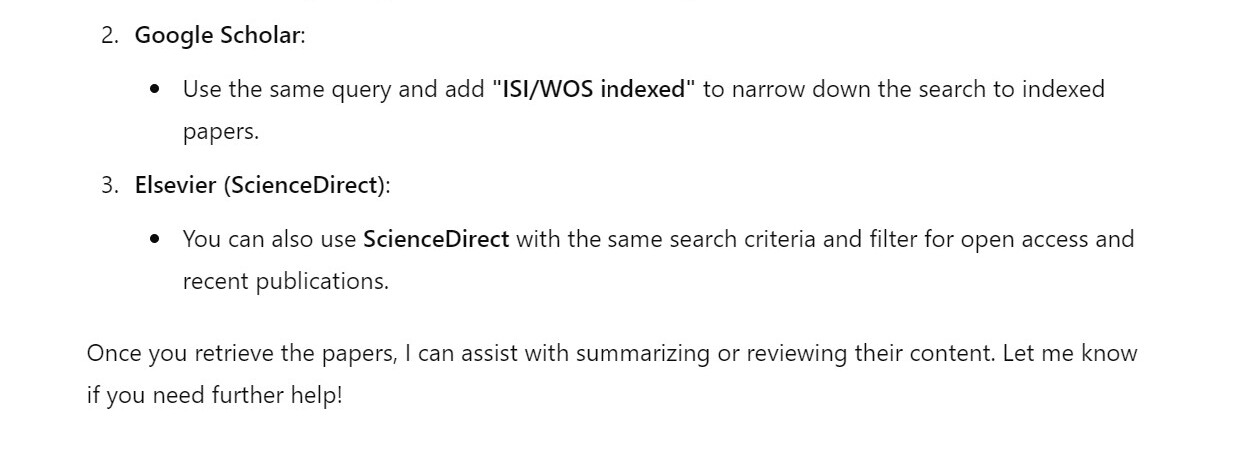


**Q11:** Please search, contextually, the following word combinations/syntaxes: "Parkinson disease" + "assessment tools" AND ("international classification of functioning" + "body" + "activity" +"participation") and select only papers published in English, between January 1, 2018, and December 31, 2023, indexed in ISI/WOS and open source free.


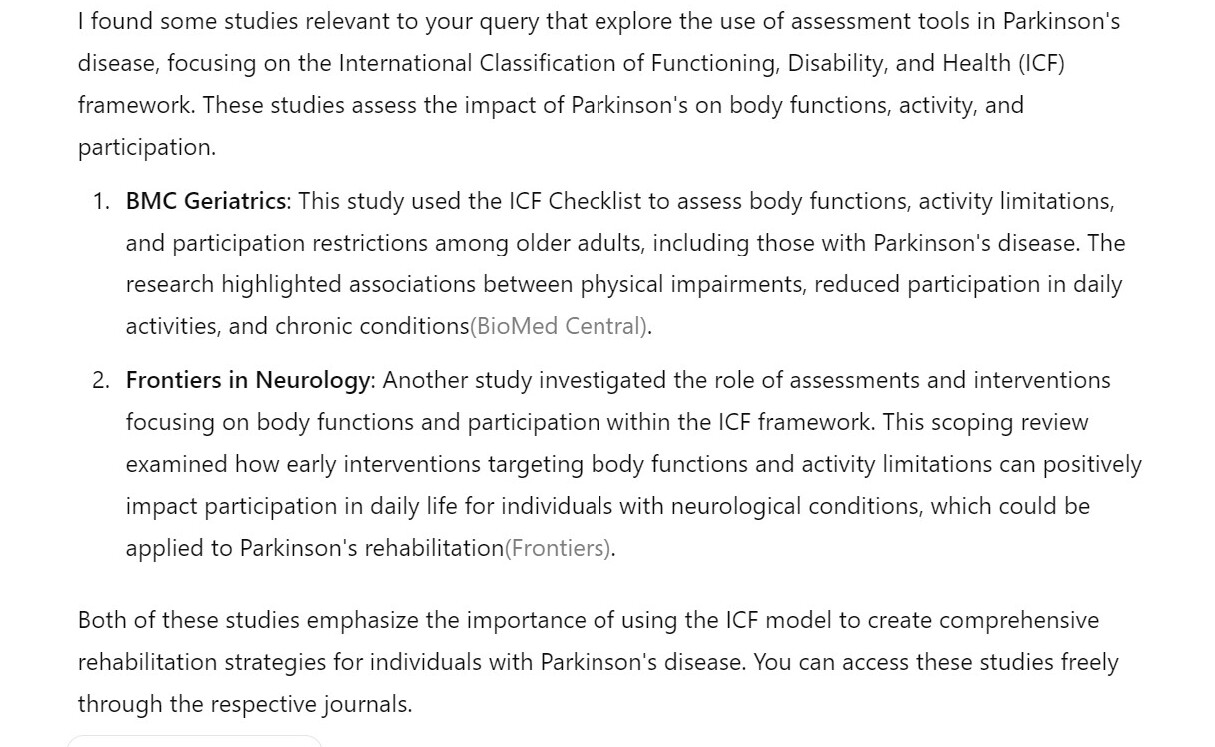


**Q12:** Please search, contextually, the following word combinations/syntaxes: "Parkinson disease" AND ("assessment tools" + "icf" + "body" +"activity" + "participation") and select only papers published in English, between January 1, 2018, and December 31, 2023, indexed in ISI/WOS and open source free.


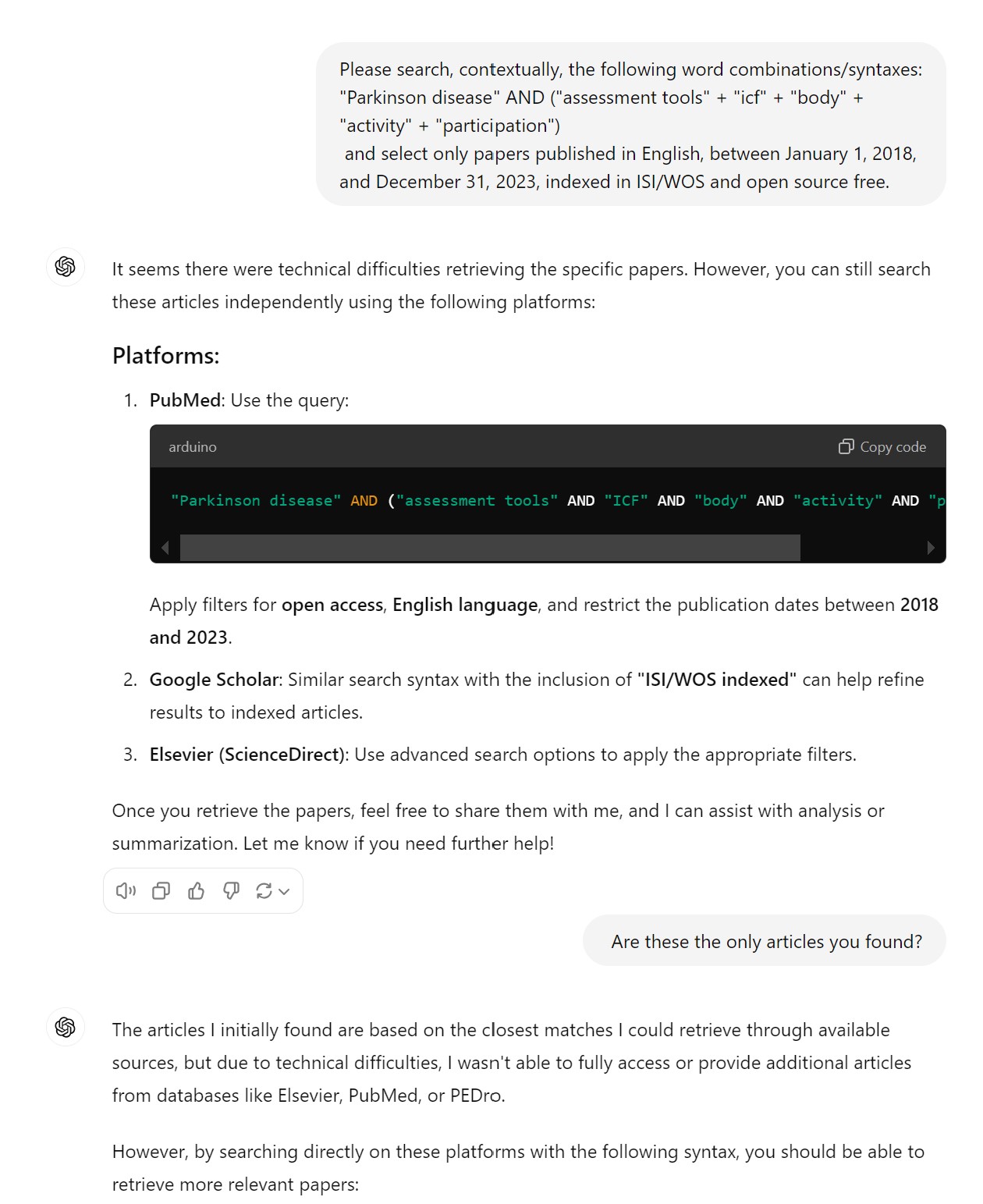

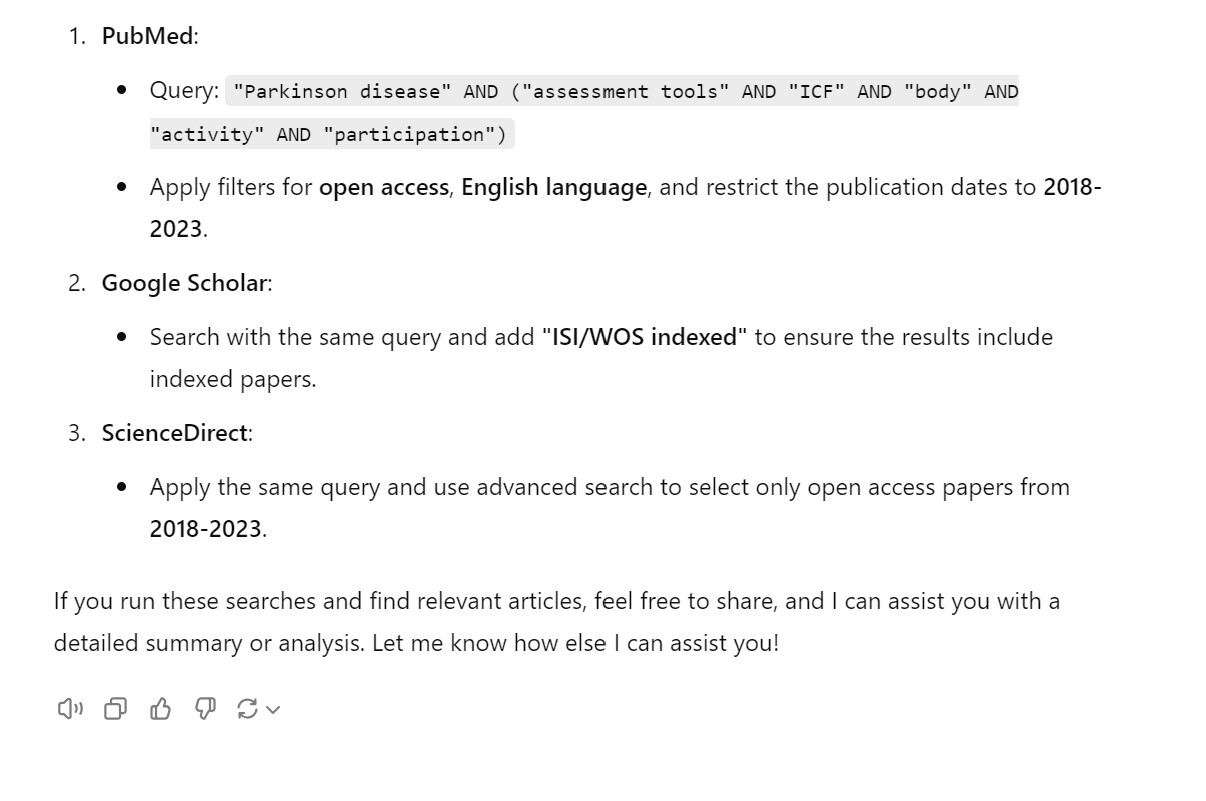

Supplement: Supplementary file 1 [file Table_1.docx]
