# Supplementary material for: Standardized clinical assessments and advanced AI-driven instruments used to evaluate neurofunctional deficits, including within biomarker based framework, in Parkinson’s disease - human intelligence made vs. AI models - systematic review
Source: Front Med (Lausanne). 2025 Jun 13;12:1565275. doi: 10.3389/fmed.2025.1565275 (PMC12202485; doi:10.3389/fmed.2025.1565275)
Supplement: Supplementary file 2 [file Table_2.docx]

Legend: **Red** = wrong answer. **Green** = correct(meeting the search criteria by syntaxes) but**,** was published out of the period within our PRISMA type systematic literature review(2010 Jul).

.

***Q1****: Indicate academic papers (title, authors, DOI) referring to the following keywords:. "Parkinson disease " + "assessment scales" + "international classification of functioning" + "body" + "activity" + "participation”*

**The first GPT4.0 interrogation**

a)**"Physical Therapist Management of Parkinson Disease: A Model of Care**" Cynthia L. Bertram, Diane M. Damiano, et al.DOI: 10.1093/ptj/pzab302

b)**"The Impact of Clinical Scales in Parkinson’s Disease: A Systematic Review"** Magnus H. Nilsson, Anne Westergren, et al. DOI: 10.1186/s12883-020-01996-7

c)**"Uncovering Indicators of the International Classification of Functioning, Disability, and Health from the 39-item Parkinson’s Disease Questionnaire"** Carlsson G., Hagell P., et al. DOI: 10.4061/2010/984673

d)**"Parkinson Disease: Current Assessment Methods and Wearable Devices for Evaluation of Movement Disorder Motor Symptoms"** AlMahadin G., Lotfi A., et al. DOI: 10.1186/s12883-020-01996-7

e) **"Parkinson Evidence Database to Guide Effectiveness (PDEDGE) Task Force"** URL: www.neuropt.org

**The second GPT4.0 interrogation**

a)**"Validation of the Comprehensive ICF Core Set for Parkinson's Disease: An International Multicenter Study"** Cieza A, Stucki G, Weigl M, Kollerits B, Chatterji S, Ustün BT, Kostanjsek N. **DOI**: 10.1016/j.parkreldis.2006.05.003 b)**"The Use of ICF in Assessing the Impact of Parkinson's Disease on Functioning and Disability"** Leonardi M, Raggi A, Pagani M, Carella F, Soliveri P, Albanese A. **DOI**: 10.1016/j.parkreldis.2008.01.004

c)**"ICF Core Sets for Parkinson's Disease: Validation and Clinical Utility"** Stier-Jarmer M, Grill E, Ewert T, Bartholomeyczik S, Finger M, Mokrusch T, Wessel K, Stucki G. **DOI**: 10.2340/16501977-0034. d) **"A Systematic Review of ICF-Based Assessment Instruments for Parkinson's Disease: Focus on Body Functions, Activities, and Participation"** Müller B, Assmus J, von Campenhausen S, Stolze H, Riedel O, Dodel R. **DOI**: 10.1016/j.parkreldis.2009.05.001.

e) **"ICF and Assessment Scales for Parkinson’s Disease: An Integration of Body Functions, Activities, and Participation"** Grimby G, Stibrant Sunnerhagen K. **DOI**: 10.2340/16501977-0010

***Q2****: Indicate academic papers (title, authors, DOI) referring to the following keywords: Parkinson disease + "assessment scales" + "icf" + "body" + "activity" + "participation"*

**The first GPT4.0 interrogation**

**a)"Physical Therapist Management of Parkinson Disease: A Model of Care"** Cynthia L. Bertram, Diane M. Damiano, et al.**DOI:** 10.1093/ptj/pzab302

**b)"The Impact of Clinical Scales in Parkinson’s Disease: A Systematic Review"** Magnus H. Nilsson, Anne Westergren, et al. **DOI:**10.1186/s12883-020-01996-7.

**c) "Uncovering Indicators of the International Classification of Functioning, Disability, and Health from the 39-item Parkinson’s Disease Questionnaire"** Carlsson G., Hagell P., et al. **DOI**: 10.4061/2010/984673.

d) **"Parkinson Disease: Current Assessment Methods and Wearable Devices for Evaluation of Movement Disorder Motor Symptoms"** AlMahadin G., Lotfi A., et al. **DOI**: 10.1186/s12883-020-01996-7.

**e) "Parkinson Evidence Database to Guide Effectiveness (PDEDGE) Task Force"** URL: www.neuropt.org

**The second GPT4.0 interrogation**

a)**"Validation of the Comprehensive ICF Core Set for Parkinson's Disease: An International Multicenter Study"** Cieza A, Stucki G, Weigl M, Kollerits B, Chatterji S, Ustün BT, Kostanjsek N. **DOI**: 10.1016/j.parkreldis.2006.05.003

b)**"ICF Core Sets for Parkinson's Disease: An International Consensus Process"** Stier-Jarmer M, Grill E, Ewert T, Bartholomeyczik S, Finger M, Mokrusch T, Wessel K, Stucki G. **DOI**: 10.2340/16501977-0034

c)**"Using the ICF Framework to Assess Disability in Parkinson's Disease: Development of a Disease-Specific ICF Core Set"** Leonardi M, Raggi A, Pagani M, Carella F, Soliveri P, Albanese A. **DOI**: 10.1002/mds.21279

d)**"Development and Validation of an ICF-Based Assessment Tool for Parkinson’s Disease"** Müller B, Assmus J, von Campenhausen S, Stolze H, Riedel O, Dodel R. **DOI**: 10.1016/j.parkreldis.2009.05.001

e)**"Assessment of Functioning and Disability in Patients with Parkinson’s Disease Using the ICF Framework"** Grimby G, Stibrant Sunnerhagen K. **DOI**: 10.2340/16501977-0010

***Q3:*** *Indicate academic papers (title, authors, DOI) referring to the following keywords: "PD" + "assessment scales" + "international classification of functioning" + "body" + "activity" + "participation"*

**The first GPT4.0 interrogation**

**a) "Uncovering indicators of the International Classification of Functioning, Disability, and Health from the 39-item Parkinson's disease questionnaire"** Nilsson MH, Westergren A, Carlsson G, Hagell P. **DOI**: 10.4061/2010/984673.

**b) "The impact of clinical scales in Parkinson’s disease: a systematic review"** van Wamelen DJ, Martinez‐Martin P, Weintrau D, Schrag A, Antonini A, et al. **DOI**: 10.1186/s12883-020-01996-7

**c) "Mapping the International Classification of Functioning, Disability, and Health (ICF) to Parkinson’s Disease: Developing Core Sets"** Geyh S, Kurt T, Cieza A, Stucki G. **DOI**: 10.1177/1545968307311107.

**d) "The ICF Core Sets for Parkinson's Disease: Validation of the Activities and Participation Domain"** Müller B, Assmus J, Herlofson K, Larsen JP, Peto V.**DOI**: 10.1016/j.parkreldis.2010.06.011

**The second GPT4.0 interrogation**

a) **"The International Classification of Functioning, Disability and Health: A New Tool for Understanding Disability and Health"** Üstün TB, Chatterji S, Kostanjsek N, Rehm J, Kennedy C, Epping-Jordan J, Saxena S, Von Korff M, Pull C. **DOI**: 10.1016/S0140-6736(03)13877-1

b) **"The International Classification of Functioning, Disability and Health (ICF) Core Sets for Hand Conditions"** Stamm TA, Cieza A, Machold K, Smolen J, Stucki G DOI: 10.1097/01.bor.0000210943.01025.3d

c) "**Application of the ICF in clinical practice"** Rauch A, Cieza A, Stucki G **DOI**: 10.1007/s00391-008-0501-5

d) "**The ICF: Applications of the WHO model of functioning, disability and health to brain injury rehabilitation"** Tate R, Kennedy M, Ponsford J, Douglas J, Velikonja D, Bayley M, Stergiou-Kita M **DOI**: 10.1080/09602011.2013.823370

e)" **Measurement properties of the International Classification of Functioning, Disability and Health core sets for post-acute stroke**" Geyh S, Cieza A, Schouten J, Dickson H, Frommelt P, Omar Z, Kostanjsek N, Ring H, Stucki G **DOI**: 10.2340/16501977-0034

***Q4****: Indicate academic papers (title, authors, DOI) referring to the following keywords: "PD" + "assessment scales" + "international classification of functioning" + "body" + "activity" + "participation"*

**The first GPT4.0 interrogation**

a)**"Mapping the International Classification of Functioning, Disability and Health to Parkinson's Disease: Developing Core Sets"** Geyh S, Kurt T, Cieza A, Stucki G.DOI: 10.1177/1545968307311107.

b) **"Uncovering Indicators of the International Classification of Functioning, Disability, and Health from the 39-item Parkinson's Disease Questionnaire"** Nilsson MH, Westergren A, Hagell P. DOI: 10.4061/2010/984673

c) **"The ICF Core Sets for Parkinson's Disease: Validation of the Activities and Participation Domain"** Müller B, Assmus J, Herlofson K, Larsen JP, Peto V. DOI: 10.1016/j.parkreldis.2010.06.011.

d) **"The Impact of Clinical Scales in Parkinson’s Disease: A Systematic Review**" van Wamelen DJ, Martinez‐Martin P, Weintraub D, Schrag A, Antonini A, et al. DOI: 10.1186/s12883-020-01996-7

e)**"Health-Related Quality of Life and Disability in Parkinson's Disease: A Comparison of the EQ-5D-5L and the ICF Framework"** Hiller AP, Panteli M, Giannouli E, Dimitrova D, Herman T, et al. DOI: 10.1016/j.jns.2020.116690

**The second GPT4.0 interrogation**

a) **“ICF core sets for the assessment of Parkinson's disease: results of the international consensus process"** Stier-Jarmer M, Grill E, Ewert T, Bartholomeyczik S, Finger M, Mokrusch T, Wessel K, Stucki G. DOI: 10.2340/16501977-0034

b)**"Development and validation of an ICF-based disability measure for Parkinson's disease: the ICF-PD"** Klingelhoefer L, Chaudhuri KR, Ashkan K, Samuel M. **DOI**: 10.1002/mds.27061

c)**"Assessing disability in people with Parkinson's disease: comparison of the UPDRS and the ICF framework"**  Üstün TB, Chatterji S, Kostanjsek N, Rehm J, Kennedy C, Epping-Jordan J. **DOI**: 10.1002/mds.20956

d) **"Integrating the ICF into the clinical management of Parkinson's disease: benefits and challenges"** Schrag A, Selai C, Jahanshahi M, Quinn NP. **DOI**: 10.1016/j.parkreldis.2007.09.005

***Q5****: Indicate academic papers (title, authors, DOI) referring to the following keywords: "Parkinson disease " + "assessment instruments" + "international classification of functioning" + "body" + "activity" + "participation"*

**The first GPT4.0 interrogation**

a)**"Applying the International Classification of Functioning, Disability, and Health (ICF) to Assess Parkinson's Disease: Development and Validation of Core Sets"** Geyh S, Kurt T, Cieza A, Stucki G.DOI: 10.1016/j.parkreldis.2004.09.006.

b) **"Clinimetric Evaluation of Instruments Used to Assess Functioning in Parkinson's Disease: Applying the ICF Framework"** Metral M, Laffont I, Schnitzler A. DOI: 10.1016/j.apmr.2020.06.017.

c) **"ICF Core Sets for Parkinson’s Disease: Linking Disease-Specific Assessment Instruments to the ICF**" Grimbergen YA, Munneke M, Bloem BR. DOI: 10.1016/j.parkreldis.2010.06.012.

d) **"The ICF Framework and Parkinson's Disease: Linking the ICF Core Sets to Clinical Assessment Instruments"** van der Marck MA, Klok MP, Okun MS, Munneke M, Bloem BR. DOI: 10.1007/s00415-014-7188-9.

f) "**Validation of the Comprehensive ICF Core Sets for Parkinson’s Disease: An Analysis of Assessment Instruments"** Müller B, Assmus J, Herlofson K, Larsen JP, Peto V. DOI: 10.1016/j.parkreldis.2011.02.014

**The second GPT4.0 interrogation**

a) **"The use of ICF in identifying factors influencing activity and participation in persons with Parkinson's disease"** Leonardi M, Raggi A, Pagani M, Carella F, Soliveri P, Albanese A. **DOI**: 10.1002/mds.21279

b) **"Development and Validation of an ICF-Based Disability Measure for Parkinson's Disease: The ICF-PD"** Klingelhoefer L, Chaudhuri KR, Ashkan K, Samuel M. **DOI**: 10.1002/mds.27061

c) **"ICF core sets for the assessment of Parkinson's disease: Results of the international consensus process"** Stier-Jarmer M, Grill E, Ewert T, Bartholomeyczik S, Finger M, Mokrusch T, Wessel K, Stucki G. **DOI**: 10.2340/16501977-0034

d) **"The International Classification of Functioning, Disability and Health (ICF) as a framework for identifying and measuring functioning and disability in Parkinson's disease"** Üstün TB, Chatterji S, Kostanjsek N, Rehm J, Kennedy C, Epping-Jordan J. **DOI**: 10.1002/mds.20956

e)**"Assessing disability and quality of life in Parkinson's disease: Validation of the ICF core sets"** Schrag A, Selai C, Jahanshahi M, Quinn NP. **DOI**: 10.1016/j.parkreldis.2007.09.005

***Q6****: Indicate academic papers (title, authors, DOI) referring to the following keywords: Parkinson disease " + "assessment instruments" + "icf" + "body" + "activity" + "participation"*

**The first GPT4.0 interrogation**

a)**"The Use of ICF in Identifying Problems in Functioning of People with Parkinson's Disease"**: Grimbergen YA, Schrag A, Mazibrada G, Borm GF, Bloem BR. **DOI**: 10.1016/j.parkreldis.2006.05.008. b)"**ICF Core Sets for Parkinson's Disease: Assessment of Body Functions, Activities, and Participation"** Müller B, Assmus J, Herlofson K, Larsen JP, Peto V. **DOI**: 10.1016/j.parkreldis.2008.04.014. c)**"Assessing Participation in People with Parkinson’s Disease: A Study Using the ICF Framework"** Cieza A, Stucki G, Weigl M, Disler P, Jäckel WH. **DOI**: 10.1080/09638280400014886. d) **"Evaluating Disability in Parkinson's Disease with the International Classification of Functioning, Disability and Health (ICF)"** Schenkman M, Ellis T, Christiansen C, Barón AE, Nevit M. **DOI**: 10.1016/j.apmr.2010.06.010. e) **"Validation of ICF Core Sets for Use in Parkinson's Disease: Linking Assessment Instruments to ICF Categories"** van der Marck MA, Bloem BR, Munneke M. **DOI**: 10.1007/s00415-012-6603-0

**The second GPT4.0 interrogation**

a) **"ICF Core Sets for Parkinson’s Disease: An International Approach to Specify Functioning"** Stier-Jarmer M, Grill E, Ewert T, Bartholomeyczik S, Finger M, Mokrusch T, Wessel K, Stucki **DOI**: 10.2340/16501977-0034

b) **"Application of the ICF in clinical practice for Parkinson's disease: Assessment instruments and tools"** Leonardi M, Raggi A, Pagani M, Carella F, Soliveri P, Albanese A. **DOI**: 10.1002/mds.21279

c) **"Using the International Classification of Functioning, Disability, and Health to Understand Disability in Parkinson's Disease"** Üstün TB, Chatterji S, Kostanjsek N, Rehm J, Kennedy C, Epping-Jordan J. **DOI**: 10.1002/mds.20956

d) **"Validation of ICF Core Sets for Parkinson's Disease: Assessing Body Functions, Activities, and Participation"** Schrag A, Selai C, Jahanshahi M, Quinn NP **DOI**: 10.1016/j.parkreldis.2007.09.005

e) **"The ICF Framework in Parkinson's Disease: Integrating Assessment Instruments for a Comprehensive Evaluation"** Klingelhoefer L, Chaudhuri KR, Ashkan K, Samuel M **DOI**: 10.1002/mds.27061

***Q7****: Indicate academic papers (title, authors, DOI) referring to the following keywords: "PD" + "assessment instruments" + "international classification of functioning" + "body" + "activity" + "participation"*

**The first GPT4.0 interrogation**

a)**"The International Classification of Functioning, Disability and Health (ICF) Core Sets for Parkinson’s Disease: Results of the Validation Process"** Geyh S, Kurt T, Cieza A, Stucki G. DOI: 10.1016/j.parkreldis.2004.09.006.

b) **"Linking Parkinson’s Disease Assessment Instruments to the International Classification of Functioning, Disability, and Health (ICF): An Exploratory Study"** Grimbergen YA, Schrag A, Mazibrada G, Borm GF, Bloem BR. DOI: 10.1016/j.parkreldis.2006.05.008.

c)**"Assessing Disability and Health in Parkinson’s Disease Using the International Classification of Functioning, Disability, and Health (ICF)"**Cieza A, Stucki G, Weigl M, Disler P, Jäckel WH. DOI: 10.1080/09638280400014886.

d) "**Validation of ICF Core Sets for Parkinson’s Disease: Linking Assessment Instruments to the ICF"** van der Marck MA, Munneke M, Bloem BR. DOI: 10.1007/s00415-012-6603-0.

e) **"Using the ICF to Assess Functioning and Disability in Parkinson’s Disease: An Analysis of Available Assessment Instruments"** Müller B, Assmus J, Herlofson K, Larsen JP, Peto V. DOI: 10.1016/j.parkreldis.2011.02.014

**The second GPT4.0 interrogation**

a) **"The Comprehensive ICF Core Set for Parkinson's Disease: A Validation Study"** Stier-Jarmer M, Grill E, Ewert T, Bartholomeyczik S, Finger M, Mokrusch T, Wessel K, Stucki G. **DOI**: 10.2340/16501977-0034

b)**"Measuring Disability in Parkinson's Disease: A Comparison of the UPDRS and the ICF Framework"** Leonardi M, Raggi A, Pagani M, Carella F, Soliveri P, Albanese A. **DOI**: 10.1002/mds.21279

c)**"ICF Core Sets for Parkinson's Disease: An International Consensus Process"** Müller B, Assmus J, von Campenhausen S, Stolze H, Riedel O, Dodel R. **DOI**: 10.1016/j.parkreldis.2009.05.001

d) **"Validation of the ICF Core Set for Patients with Parkinson’s Disease: A Multicenter Study"** Cieza A, Brockow T, Ewert T, Amann E, Kollerits B, Chatterji S, Ustün BT, Stucki G. **DOI**: 10.1191/0269215505cr899oa

e)**"Assessment of Functioning and Disability in Patients with Parkinson’s Disease Using the ICF Framework"** Grimby G, Stibrant Sunnerhagen K. DOI: 10.2340/16501977-0010

***Q8****: Indicate academic papers (title, authors, DOI) referring to the following keywords : "PD" + "assessment instruments" + "icf" + "body" + "activity" + "participation"*

**The first GPT4.0 interrogation**

a) **"Developing Core Sets for Parkinson's Disease Based on the International Classification of Functioning, Disability, and Health (ICF)"** Müller B, Assmus J, Herlofson K, Larsen JP, Peto V. DOI: 10.1016/j.parkreldis.2008.04.014 b) **"The Role of the ICF in the Assessment of Disability and Health in Patients with Parkinson’s Disease"** Cieza A, Geyh S, Chatterji S, Kostanjsek N, Ustün BT, Stucki G. DOI: 10.1016/j.parkreldis.2006.06.008 c) **"Validation of ICF Core Sets for Parkinson's Disease: A Focus on Assessment Instruments for Body Functions, Activities, and Participation"** Grimbergen YA, Munneke M, Bloem BR. DOI: 10.1007/s00415-010-5653-5. d) **"Assessing Parkinson's Disease with the ICF: Validation of the Comprehensive ICF Core Sets and Assessment Instruments"** van der Marck MA, Klok MP, Okun MS, Munneke M, Bloem BR. DOI: 10.1016/j.parkreldis.2012.05.010. e) **"Linking Assessment Instruments to the ICF: An Analysis in Parkinson's Disease"** Nilsson MH, Hagell P. DOI: 10.1016/j.parkreldis.2013.11.013

**The second GPT4.0 interrogation**

a)**"ICF Core Sets for Parkinson’s Disease: An International Approach to Specify Functioning"** Stier-Jarmer M, Grill E, Ewert T, Bartholomeyczik S, Finger M, Mokrusch T, Wessel K, Stucki G. DOI: 10.2340/16501977-0034

b)**"The Development of an ICF-Based Instrument to Measure Activity and Participation in Parkinson's Disease"**Müller B, Assmus J, von Campenhausen S, Stolze H, Riedel O, Dodel R. DOI: 10.1016/j.parkreldis.2009.05.001

c) **"Using the International Classification of Functioning, Disability and Health (ICF) Framework to Measure Disability in Parkinson's Disease"** Leonardi M, Raggi A, Pagani M, Carella F, Soliveri P, Albanese A. DOI: 10.1002/mds.21279

d)**"Validation of the ICF Core Set for Patients with Parkinson’s Disease: A Multicenter Study"** Cieza A, Brockow T, Ewert T, Amann E, Kollerits B, Chatterji S, Ustün BT, Stucki G. DOI: 10.1191/0269215505cr899oa

e)**"Assessment of Functioning and Disability in Patients with Parkinson’s Disease Using the ICF Framework"** Grimby G, Stibrant Sunnerhagen K. DOI: 10.2340/16501977-0010

***Q9****: Indicate academic papers (title, authors, DOI) referring to the following keywords: "Parkinson disease " + "assessment tools" + "international classification of functioning" + "body" + "activity" + "participation’’*

**The first GPT4.0 interrogation**

a)"**ICF-Based Assessment and Classification of Functioning in Parkinson’s Disease: A Systematic Review"** van der Marck MA, Klok MP, Okun MS, Munneke M, Bloem BR. DOI: 10.1007/s00415-014-7188-9. b) "**The Comprehensive ICF Core Sets for Parkinson's Disease: Validation of the Activities and Participation Domains"** Geyh S, Kurt T, Cieza A, Stucki G. DOI: 10.1016/j.parkreldis.2004.09.006. c) **"Clinimetric Properties of Tools Assessing Functioning in Parkinson's Disease within the ICF Framework"**Metral M, Laffont I, Schnitzler A. DOI: 10.1016/j.apmr.2020.06.017. d) **"Linking the International Classification of Functioning, Disability and Health (ICF) to Parkinson's Disease Assessment Tools: A Conceptual Framework"** Grimbergen YA, Munneke M, Bloem BR. DOI: 10.1159/000104439 e) **"ICF Core Sets for Parkinson’s Disease: Validation of the Body Functions, Activities, and Participation Domains"** Müller B, Assmus J, Herlofson K, Larsen JP, Peto V. DOI: 10.1016/j.parkreldis.2011.02.014

**The second GPT4.0 interrogation**

a) **"The ICF Core Sets for Parkinson’s Disease: Validation and Clinical Application"** Stier-Jarmer M, Grill E, Ewert T, Bartholomeyczik S, Finger M, Mokrusch T, Wessel K, Stucki G.**DOI**: 10.2340/16501977-0034. b) **"Application of the ICF in Assessing Disability in Parkinson’s Disease: Tools and Methods"** Leonardi M, Raggi A, Pagani M, Carella F, Soliveri P, Albanese A. **DOI**: 10.1002/mds.21279. c) **"ICF-Based Assessment of Activity and Participation in Parkinson’s Disease: Development and Validation"** Müller B, Assmus J, von Campenhausen S, Stolze H, Riedel O, Dodel R. **DOI**: 10.1016/j.parkreldis.2009.05.001

d) **"Evaluating the Impact of Parkinson's Disease on Activities and Participation Using the ICF Framework"** Cieza A, Brockow T, Ewert T, Amann E, Kollerits B, Chatterji S, Ustün BT, Stucki G. **DOI**: 10.1191/0269215505cr899oa

e) **"Assessing Functional Limitations in Parkinson's Disease: The Role of the ICF"**  Grimby G, Stibrant Sunnerhagen K. DOI: 10.2340/16501977-0010

***Q10***: *Indicate academic papers (title, authors, DOI) referring to the following keywords: "Parkinson disease " + "assessment tools" + "icf" + "body" + "activity" + "participation"*

**The first GPT4.0 interrogation**

a)**"Development of ICF Core Sets for Parkinson's Disease: Identification of Relevant Categories of Functioning"** Geyh S, Kurt T, Cieza A, Stucki G.DOI: 10.1016/j.parkreldis.2004.09.006.

b) **"Validation of ICF Core Sets for Parkinson's Disease: A Multicenter Study"** Müller B, Assmus J, Herlofson K, Larsen JP, Peto V. DOI: 10.1016/j.parkreldis.2011.02.014 c)**"Assessment of Functioning and Disability in Patients with Parkinson’s Disease Based on the International Classification of Functioning, Disability and Health"** Nilsson MH, Westergren A, Hagell P. DOI: 10.1016/j.parkreldis.2009.05.007.

d)**"ICF-Based Assessment Tools for Evaluating Functioning in Parkinson's Disease: A Systematic Review"** van Wamelen DJ, Martinez-Martin P, Weintraub D, Schrag A, Antonini A, et al. DOI: 10.1016/j.parkreldis.2013.12.012.

e) **"The Role of the ICF in Understanding Disability and Health in Parkinson’s Disease: Assessing Body Functions, Activities, and Participation"** Alvarado-Bolaños A, Morales-Briceño H, Rodríguez-Violante M. DOI: 10.1016/j.parkreldis.2015.03.014

**The second GPT4.0 interrogation**

a)"**ICF Core Sets for Parkinson's Disease: An International Approach to Specify Functioning"** Stier-Jarmer M, Grill E, Ewert T, Bartholomeyczik S, Finger M, Mokrusch T, Wessel K, Stucki G. **DOI**: 10.2340/16501977-0034

b) **"Development and Validation of an ICF-Based Assessment Tool for Parkinson’s Disease"** Müller B, Assmus J, von Campenhausen S, Stolze H, Riedel O, Dodel R. **DOI**: 10.1016/j.parkreldis.2009.05.001

c)**"The Use of the ICF Framework in Assessing Disability in Parkinson's Disease: Tools and Techniques"** Leonardi M, Raggi A, Pagani M, Carella F, Soliveri P, Albanese A. **DOI**: 10.1002/mds.21279

d) **"ICF-Based Tools for Assessing Activity and Participation in Parkinson’s Disease: A Systematic Approach"** Cieza A, Brockow T, Ewert T, Amann E, Kollerits B, Chatterji S, Ustün BT, Stucki G. **DOI**: 10.1191/0269215505cr899oa

e) **"Functional Assessment in Parkinson's Disease Using ICF: Development of a Comprehensive Tool"** Grimby G, Stibrant Sunnerhagen K. **DOI**: 10.2340/16501977-0010

***Q11****: Indicate academic papers (title, authors, DOI) referring to the following keywords: "PD" + "assessment tools" + "international classification of functioning" + "body" + "activity" + "participation"*

**The first GPT4.0 interrogation**

a)**"Validation of the Comprehensive ICF Core Sets for Patients With Parkinson's Disease: A Multicenter Study"** Müller B, Assmus J, Herlofson K, Larsen JP, Peto V. DOI: 10.1016/j.parkreldis.2011.02.014.

b) **"Assessment of Functioning and Disability in Patients with Parkinson’s Disease Based on the International Classification of Functioning, Disability, and Health"** Geyh S, Kurt T, Cieza A, Stucki G. DOI: 10.1016/j.parkreldis.2004.09.006.

c) **"The Role of the ICF in Identifying Limitations and Facilitators in Patients with Parkinson’s Disease: A Systematic Review"** van der Marck MA, Klok MP, Okun MS, Munneke M, Bloem BR. DOI: 10.1007/s00415-014-7188-9.

d) **"Functioning and Disability in Parkinson’s Disease: The Patient Perspective Assessed by the ICF-Based Functioning Scale"** Alvarado-Bolaños A, Morales-Briceño H, Rodríguez-Violante M. DOI: 10.1007/s00415-015-7683-0.

e)**"Applying the ICF in Assessing the Outcomes of Rehabilitation in Parkinson's Disease: A Systematic Review"** Turner-Stokes L, Disler PB, Nair A, Wade DT. DOI: 10.1016/j.apmr.2005.05.018

**The second GPT4.0 interrogation**

a)"**ICF Core Sets for Parkinson's Disease: Validation and Clinical Application**" Stier-Jarmer M, Grill E, Ewert T, Bartholomeyczik S, Finger M, Mokrusch T, Wessel K, Stucki G. **DOI**: 10.2340/16501977-0034

b)**"Application of the ICF in Assessing Disability in Parkinson's Disease"** Leonardi M, Raggi A, Pagani M, Carella F, Soliveri P, Albanese A. **DOI**: 10.1002/mds.21279

c)**"Development and Validation of an ICF-Based Tool for Parkinson’s Disease"** Müller B, Assmus J, von Campenhausen S, Stolze H, Riedel O, Dodel R. **DOI**: 10.1016/j.parkreldis.2009.05.001

d)**"ICF-Based Assessment of Activity and Participation in Parkinson’s Disease"** Cieza A, Brockow T, Ewert T, Amann E, Kollerits B, Chatterji S, Ustün BT, Stucki G. **DOI**: 10.1191/0269215505cr899oa

e) **"The Role of ICF in Assessing Functionality and Disability in Parkinson's Disease"** Grimby G, Stibrant Sunnerhagen K. **DOI**: 10.2340/16501977-0010

***Q12****: Indicate academic papers (title, authors, DOI) referring to the following keywords: "PD" + "assessment tools" + "icf" + "body" + "activity" + "participation"*

**The first GPT4.0 interrogation**

a) "**ICF Core Sets for Parkinson's Disease: Validation of the Activities and Participation Domain"** Müller B, Assmus J, Herlofson K, Larsen JP, Peto V. **DOI**: 10.1016/j.parkreldis.2010.06.011. b) **"The Comprehensive ICF Core Sets for Parkinson's Disease: An Analysis of Content Validity Using the Delphi Technique"** Geyh S, Kurt T, Cieza A, Stucki G. **DOI**: 10.1016/j.parkreldis.2004.09.006. c) **"A Systematic Review of Clinimetric Properties of Functional Assessment Tools for Parkinson's Disease"** Metral M, Laffont I, Schnitzler A. DOI: 10.1016/j.apmr.2020.06.017. d) **"The Role of ICF in the Assessment of Disability and Health in Parkinson’s Disease: From Theory to Clinical Practice"** Kuopio AM, Martikainen KK, Helenius H, Toivonen M, Rinne UK. **DOI**: 10.1016/j.parkreldis.2004.08.003. e) **"Functioning and Disability in Parkinson's Disease: The Patient’s Perspective"** Alvarado-Bolaños A, Morales-Briceño H, Rodríguez-Violante M. **DOI**: 10.1016/j.parkreldis.2015.02.002.

**The second GPT4.0 interrogation**

a) **"Validation of the Comprehensive ICF Core Set for Parkinson’s Disease: A Multicenter Study"** Cieza A, Brockow T, Ewert T, Amann E, Kollerits B, Chatterji S, Ustün BT, Stucki G. **DOI**: 10.1191/0269215505cr899oa

b)"**The Use of ICF in Identifying Factors Influencing Activity and Participation in Persons with Parkinson's Disease"** Leonardi M, Raggi A, Pagani M, Carella F, Soliveri P, Albanese A.**DOI**: 10.1002/mds.21279

c)**"ICF Core Sets for Parkinson's Disease: An International Consensus Process"** Stier-Jarmer M, Grill E, Ewert T, Bartholomeyczik S, Finger M, Mokrusch T, Wessel K, Stucki G. **DOI**: 10.2340/16501977-0034

d) "**Assessing Functioning and Disability in Patients with Parkinson’s Disease Using the ICF Framework"** Grimby G, Stibrant Sunnerhagen K. **DOI**: 10.2340/16501977-0010

e)**"Development and Validation of an ICF-Based Assessment Tool for Parkinson’s Disease"** Müller B, Assmus J, von Campenhausen S, Stolze H, Riedel O, Dodel R. **DOI**: 10.1016/j.parkreldis.2009.05.001

***Results:***

***From the provided results, no article meet the search criteria. The only paper found based on the search criteria, was published out of the period within our PRISMA type systematic literature review.***
