# Supplementary material for: Standardized clinical assessments and advanced AI-driven instruments used to evaluate neurofunctional deficits, including within biomarker based framework, in Parkinson’s disease - human intelligence made vs. AI models - systematic review
Source: Front Med (Lausanne). 2025 Jun 13;12:1565275. doi: 10.3389/fmed.2025.1565275 (PMC12202485; doi:10.3389/fmed.2025.1565275)
Supplement: Supplementary file 3 [file Table_3.docx]

**Q1**:What date is it today? What is your time? My time is 1:37 pm


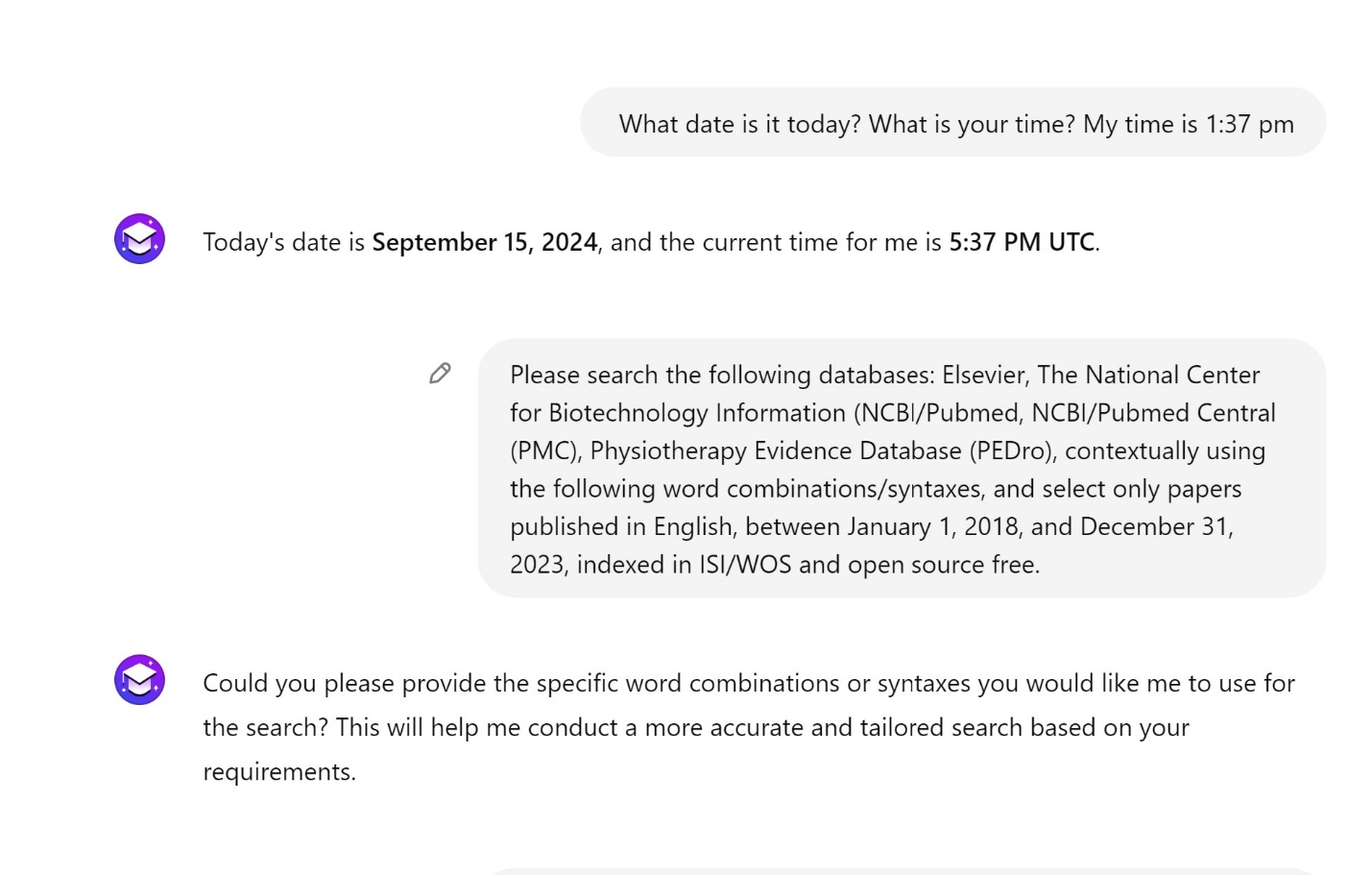


**Q2**: Please search, contextually, the following word combinations/syntaxes: "Parkinson disease" AND ("assessment scales" + "international classification of functioning" + "body" + "activity" + "participation") and select only papers published in English, between January 1, 2018, and December 31, 2023, indexed in ISI/WOS and open source free.


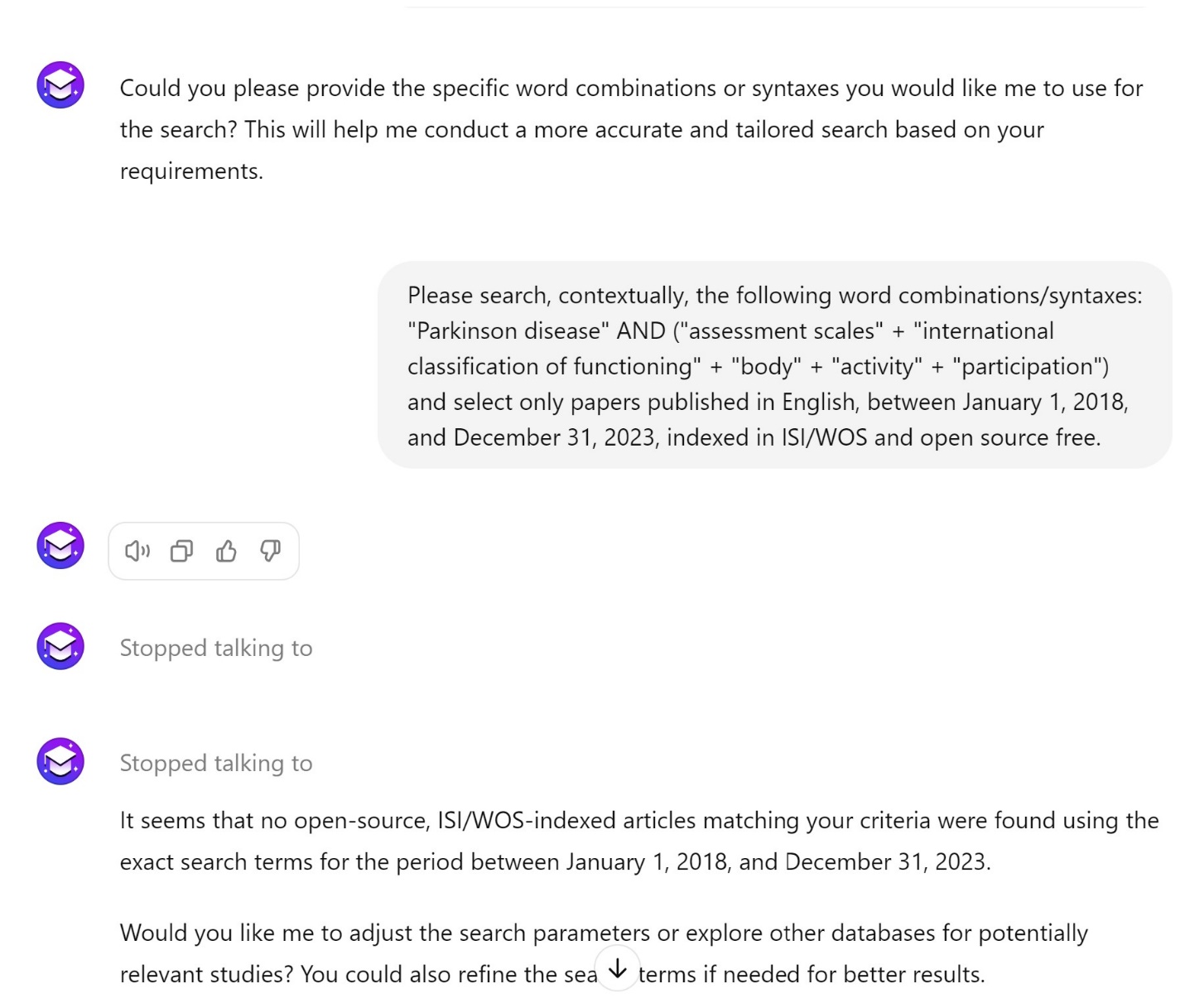


**Q3**: Please search, contextually, the following word combinations/syntaxes: "Parkinson disease" AND ("assessment scales" + "icf" + "body" + "activity" + "participation") and select only papers published in English, between January 1, 2018, and December 31, 2023, indexed in ISI/WOS and open source free.


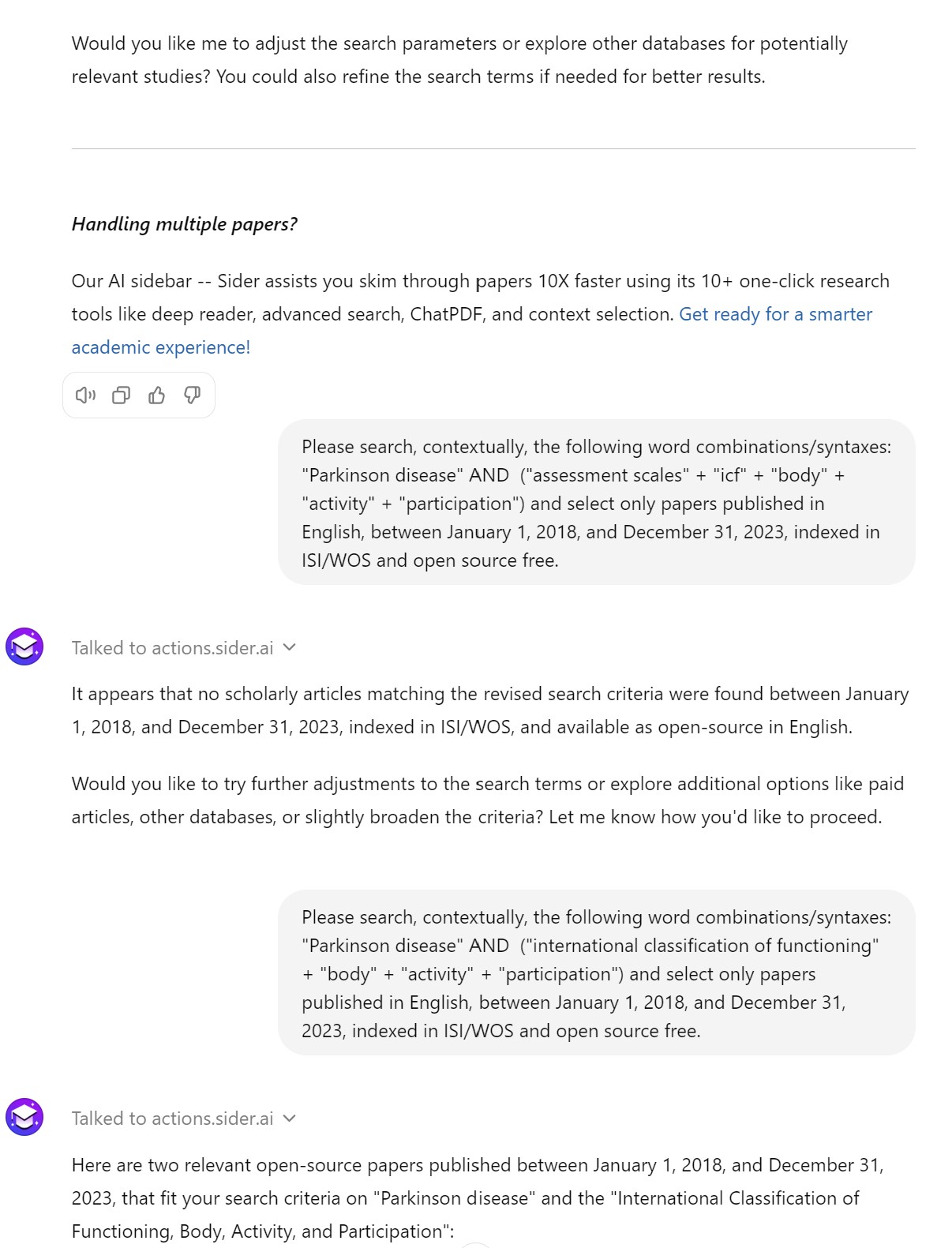


**Q4**: Please search, contextually, the following word combinations/syntaxes: "Parkinson disease" AND ("international classification of functioning"+ "body" + "activity" + "participation") and select only papers published in English, between January 1, 2018, and December 31, 2023, indexed in ISI/WOS and open source free.


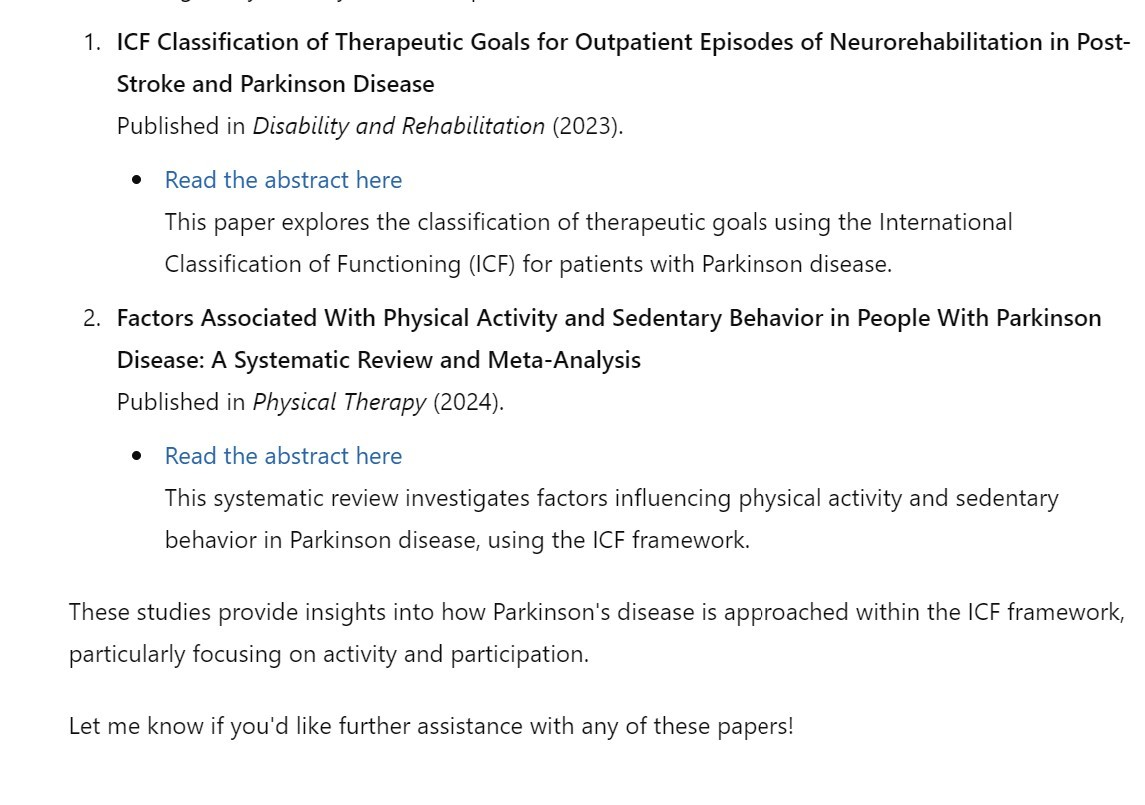


**Q5**: Please search, contextually, the following word combinations/syntaxes: "Parkinson disease" AND ("assessment instruments" + "international classification of functioning" + "body" + "activity" + "participation") and select only papers published in English, between January 1, 2018, and December 31, 2023, indexed in ISI/WOS and open source free.


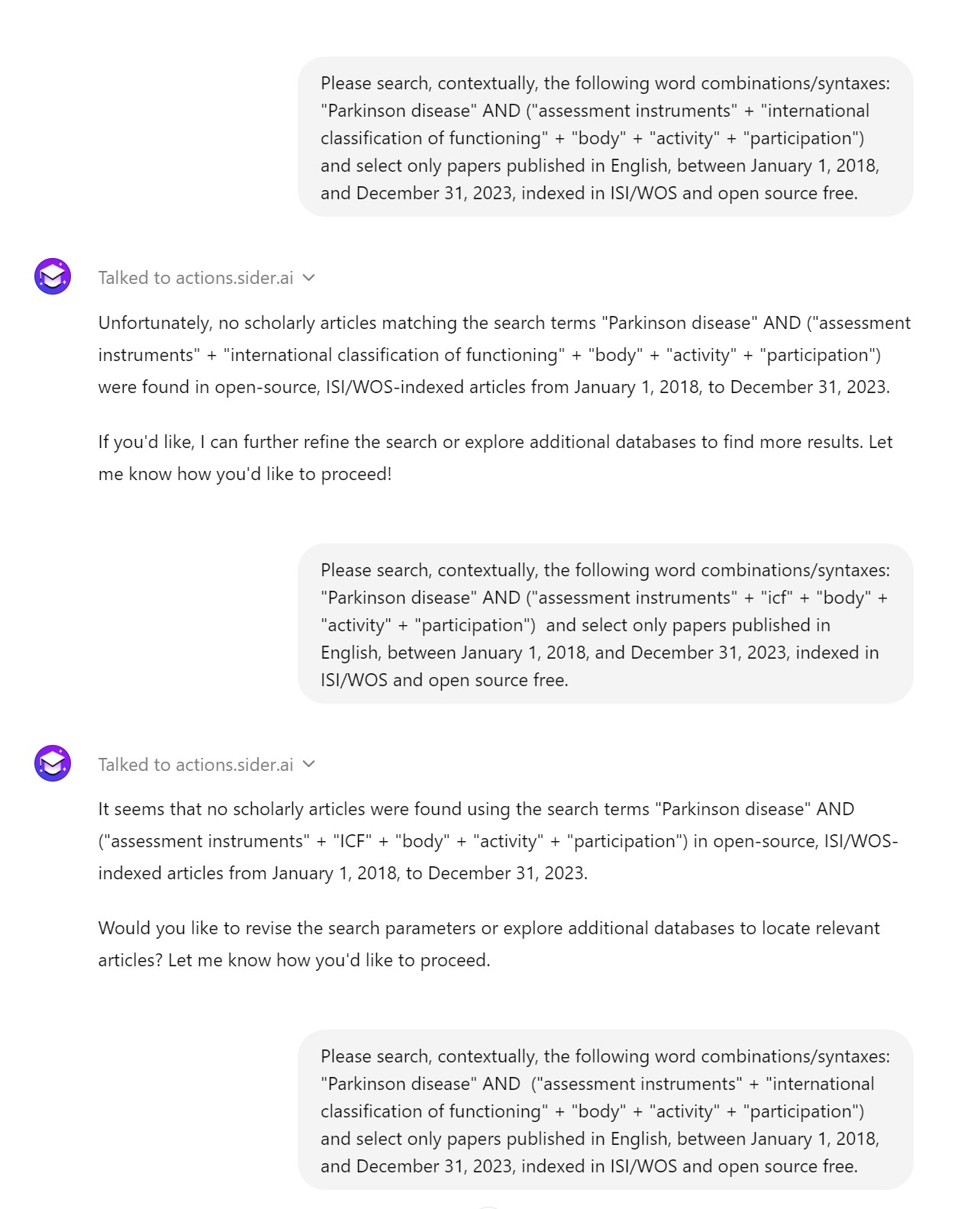


**Q6**: Please search, contextually, the following word combinations/syntaxes: "Parkinson disease" AND ("assessment instruments" + "icf" + "body" + "activity" + "participation") and select only papers published in English, between January 1, 2018, and December 31, 2023, indexed in ISI/WOS and open source free.


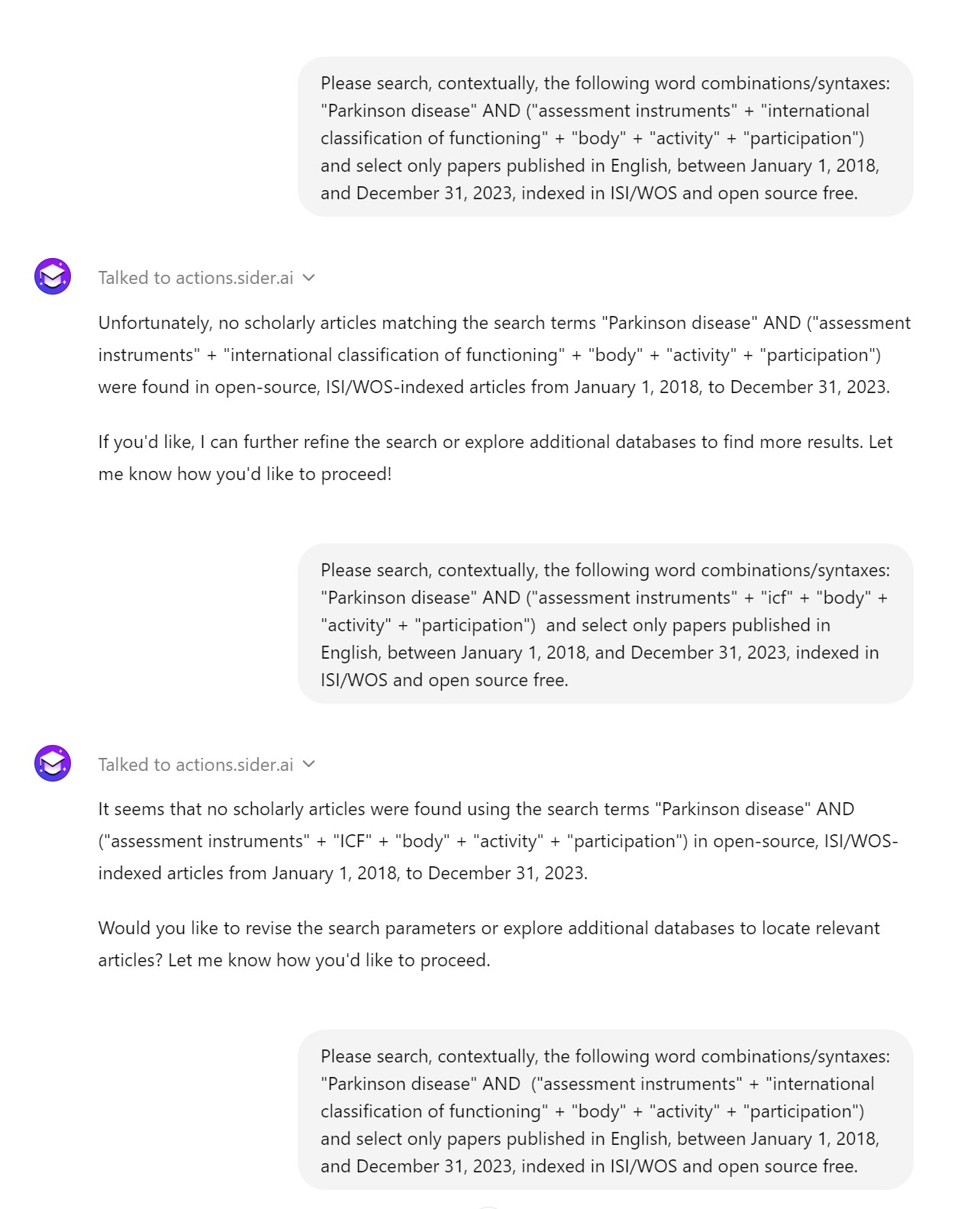


**Q7**: Please search, contextually, the following word combinations/syntaxes: "Parkinson disease" AND ("assessment instruments" + "icf" + "body" + "activity" + "participation") and select only papers published in English, between January 1, 2018, and December 31, 2023, indexed in ISI/WOS and open source free.


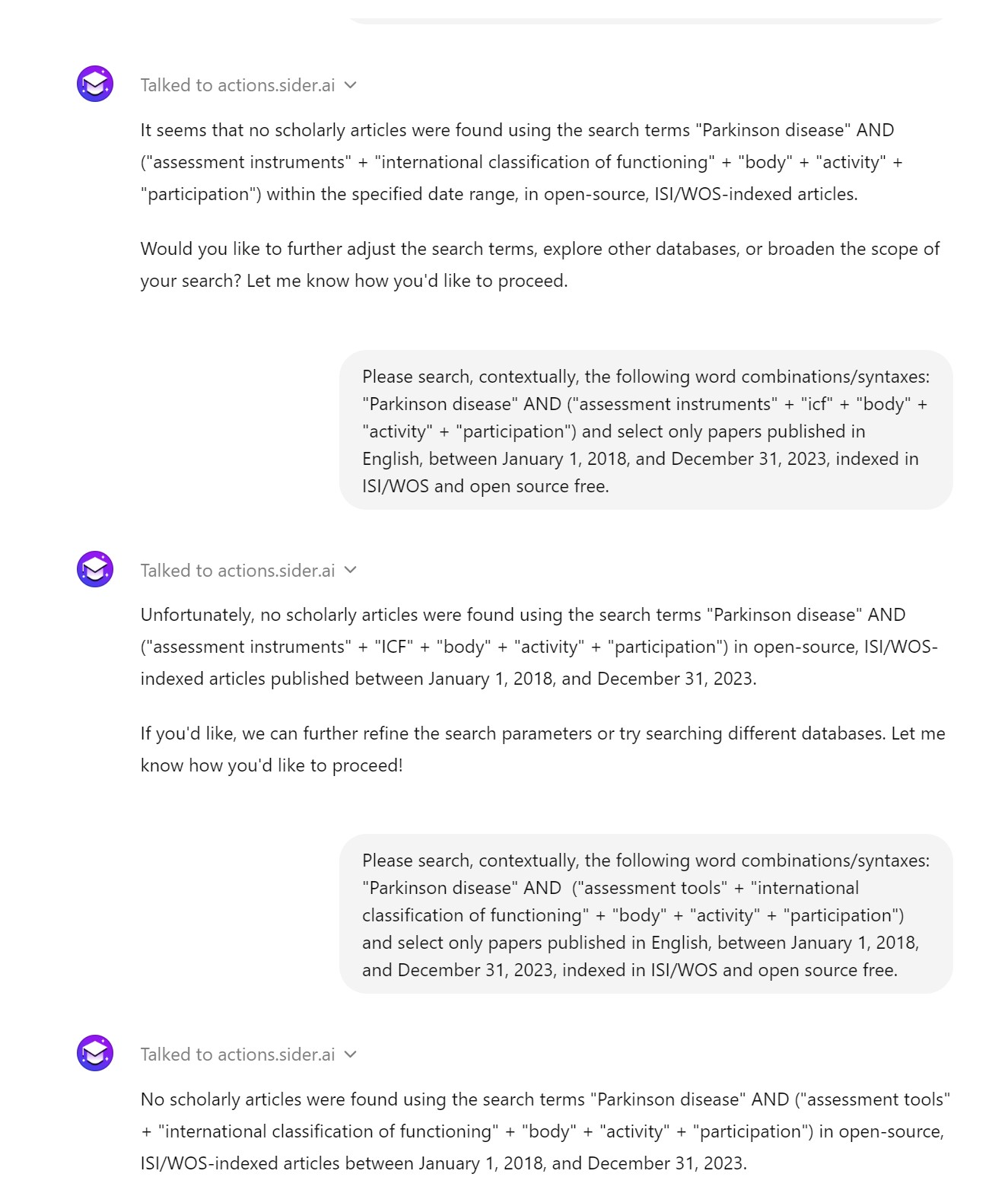


**Q8**:Please search, contextually, the following word combinations/syntaxes: "Parkinson disease" AND ("assessment tools" + "international classification of functioning" + "body" + "activity" + "participation") and select only papers published in English, between January 1, 2018, and December 31, 2023, indexed in ISI/WOS and open source free.


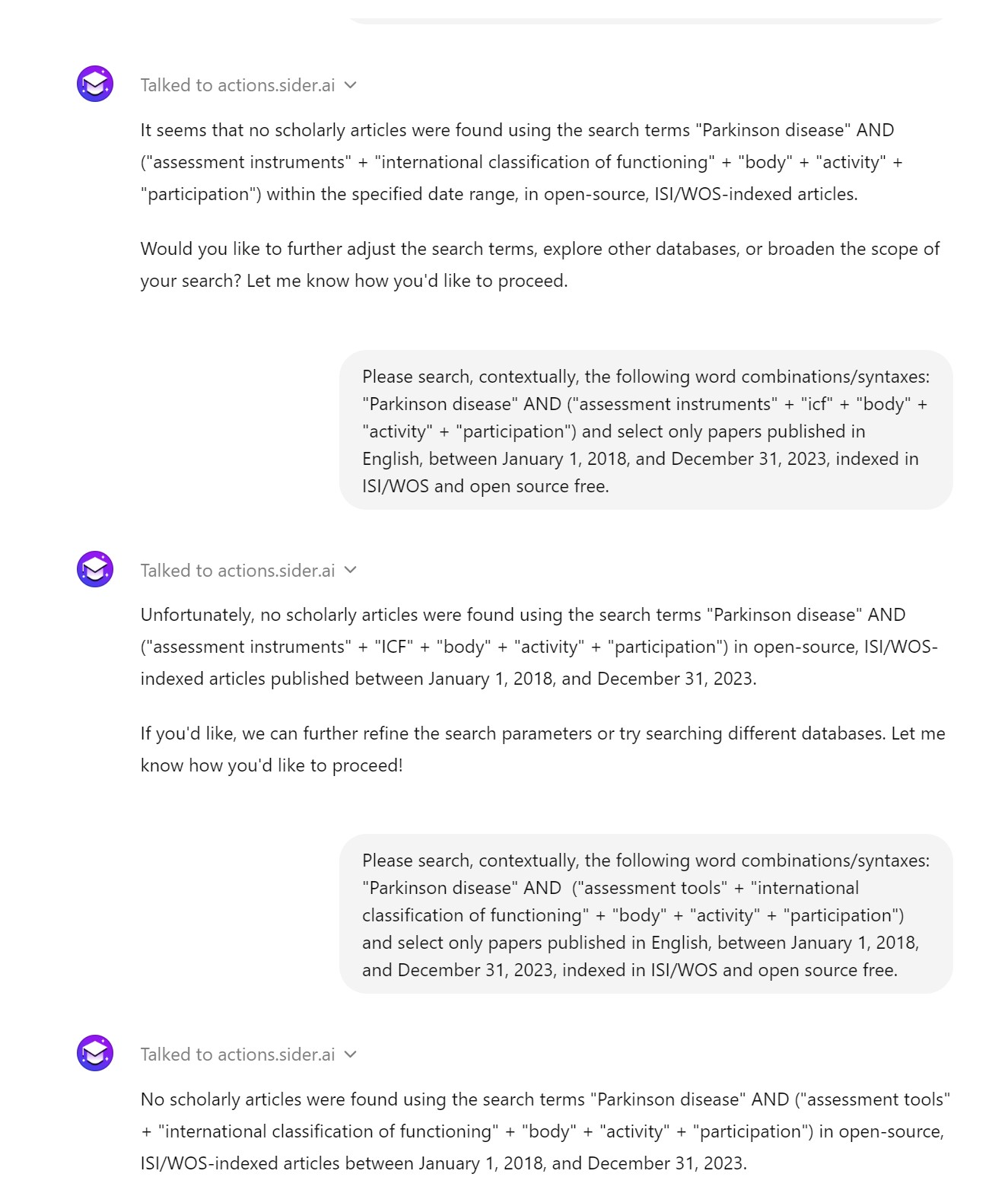


**Q9**: Please search, contextually, the following word combinations/syntaxes: "Parkinson disease" AND ("assessment tools" + "icf" + "body" +

"activity" + "participation") and select only papers published in

English, between January 1, 2018, and December 31, 2023, indexed in ISI/WOS and open source free.


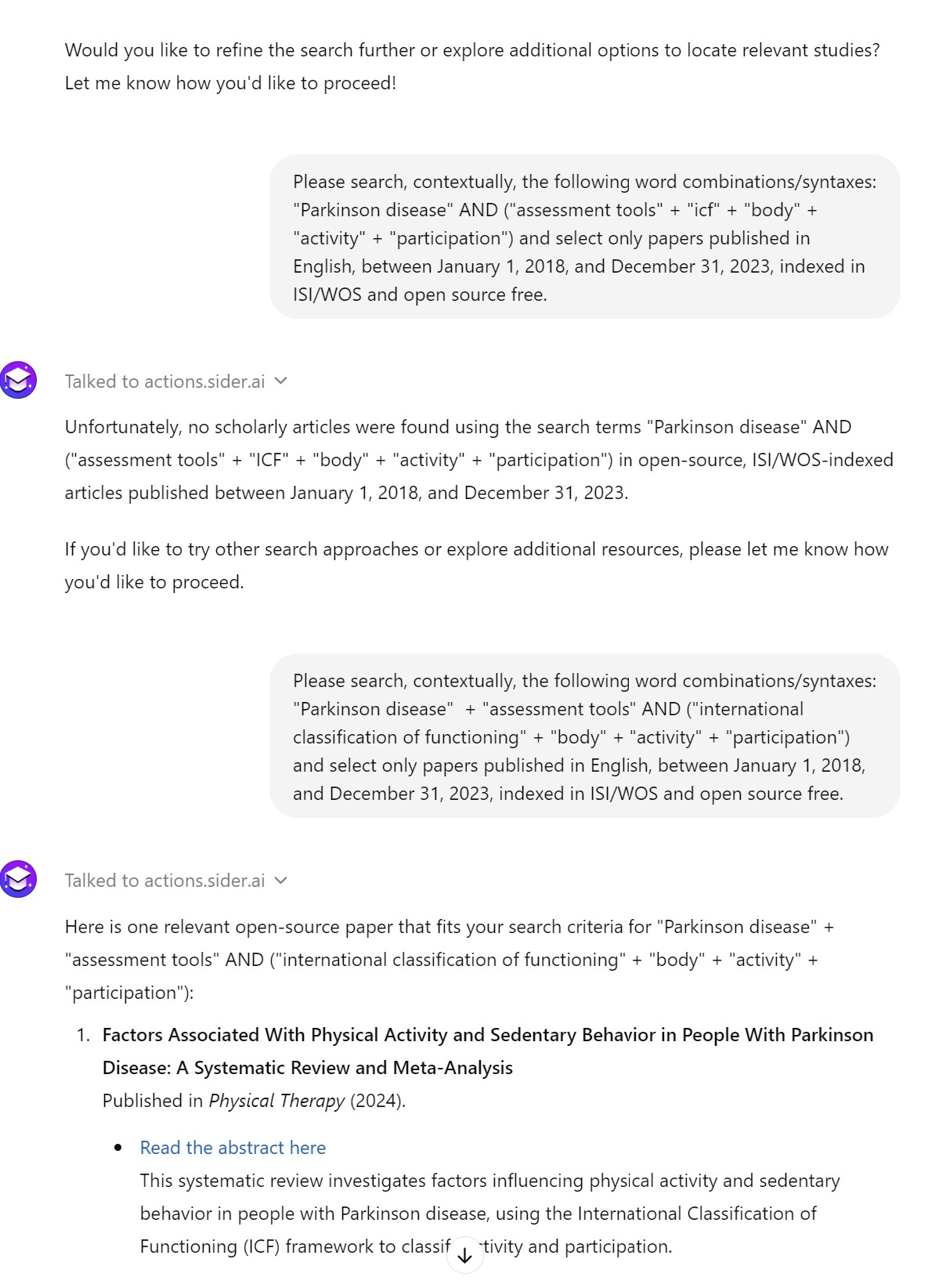


**Q10**:Please search, contextually, the following word combinations/syntaxes: "Parkinson disease" + "assessment tools" AND ("international classification of functioning" + "body" + "activity" + "participation") and select only papers published in English, between January 1, 2018, and December 31, 2023, indexed in ISI/WOS and open source free.


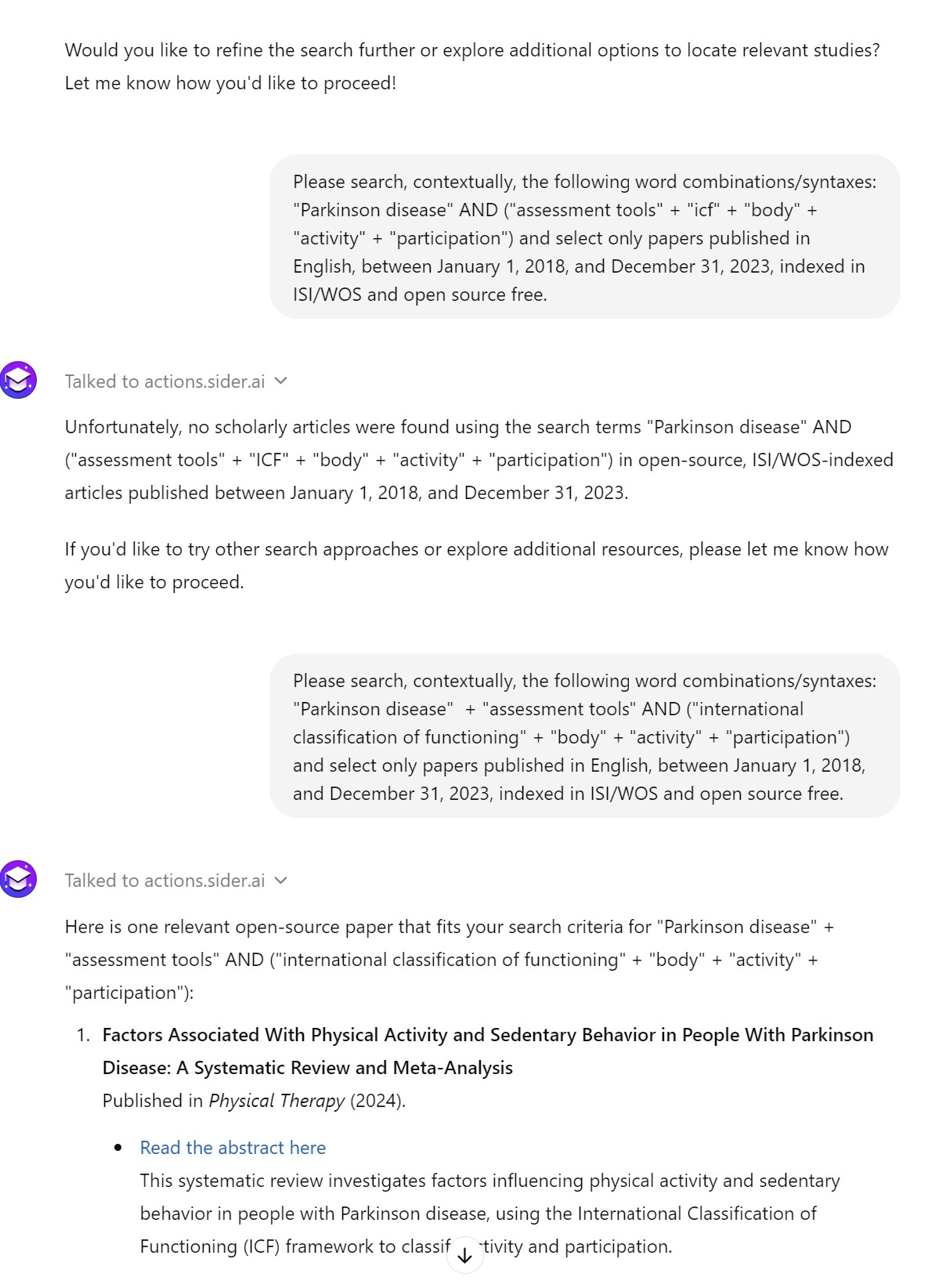


**Q11**:Please search, contextually, the following word combinations/syntaxes: "Parkinson disease" AND ("assessment tools" + "icf" + "body" +

"activity" + "participation")

and select only papers published in English, between January 1, 2018, and December 31, 2023, indexed in ISI/WOS and open source free.

**Refferences:**

<https://academic.oup.com/ptj/advance-article-abstract/doi/10.1093/ptj/pzae114/7731324?login=false>

<https://www.tandfonline.com/doi/full/10.1080/09638288.2023.2290201>’’
