# Supplementary material for: Standardized clinical assessments and advanced AI-driven instruments used to evaluate neurofunctional deficits, including within biomarker based framework, in Parkinson’s disease - human intelligence made vs. AI models - systematic review
Source: Front Med (Lausanne). 2025 Jun 13;12:1565275. doi: 10.3389/fmed.2025.1565275 (PMC12202485; doi:10.3389/fmed.2025.1565275)
Supplement: Supplementary file 4 [file Table_4.doc]

Legend: **Red** = wrong answer. **Green** = correct(meeting the search criteria by syntaxes) but unappropiated by content.

**The first general interrogation of Scholar ChatGPT**

***Q1****: Indicate academic papers (title, authors, DOI) referring to the following keywords: "Parkinson disease " + "assessment scales" + "international classification of functioning" + "body" + "activity" + "participation”*

**Scholar ChatGPT said** (Talked to actions.sider.ai)


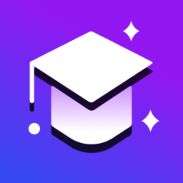


Here are academic papers related to Parkinson's disease, assessment scales, and the International Classification of Functioning (ICF):

1. **Title**: [**Scales in Parkinson's disease**](https://link.springer.com/article/10.1007/s00415-006-4008-0)
   **Authors**: G Ebersbach, H Baas, I Csoti, M Müngersdorf
   **Publication Info**: Journal of Neurology, 2006
   **DOI**: 10.1007/s00415-006-4008-0
2. **Title**: [**Evaluation of measures of upper limb functioning and disability in people with Parkinson disease: a systematic review**](https://www.sciencedirect.com/science/article/pii/S000399931401123X)
   **Authors**: EL Proud, KJ Miller, B Bilney, S Balachandran
   **Publication Info**: Archives of Physical Medicine and Rehabilitation, 2015
   **DOI**: 10.1016/j.apmr.2014.10.020
3. **Title**: [**Functioning and disability in Parkinson's disease**](https://www.tandfonline.com/doi/abs/10.3109/09638288.2010.511688)
   **Authors**: A Raggi, M Leonardi, D Ajovalasit
   **Publication Info**: Disability and Rehabilitation, 2010
   **DOI**: 10.3109/09638288.2010.511688
4. **Title**: [**Factors associated with exercise behavior in people with Parkinson disease**](https://academic.oup.com/ptj/article-abstract/91/12/1838/2735195)
   **Authors**: T Ellis, JT Cavanaugh, GM Earhart
   **Publication Info**: Physical Therapy, 2011
   **DOI**: 10.2522/ptj.20100390
5. **Title**: [**Exploring outcome measures for exercise intervention in people with Parkinson's disease**](https://onlinelibrary.wiley.com/doi/abs/10.1155/2013/572134)
   **Authors**: LA King, A Salarian, M Mancini
   **Publication Info**: Parkinson's Disease, 2013
   **DOI**: 10.1155/2013/572134
6. **Title**: [**Feasibility and informativeness of the Patient-Specific Functional Scale with patients with Parkinson's disease**](https://www.tandfonline.com/doi/shareview/10.1080/09593985.2019.1571134)
   **Authors**: RW Bohannon, P Nair, M Green
   **Publication Info**: Physiotherapy Theory and Practice, 2020
   **DOI**: 10.1080/09593985.2019.1571134
7. **Title**: [**Measuring participation as defined by the international classification of functioning, disability and health: an evaluation of existing measures**](https://www.sciencedirect.com/science/article/pii/S0003999309001191)
   **Authors**: L Resnik, MA Plow
   **Publication Info**: Archives of Physical Medicine and Rehabilitation, 2009
   **DOI**: 10.1016/j.apmr.2009.02.009
8. **Title**: [**Physical activity in patients with Parkinson's disease: a holistic approach based on the ICF model**](https://www.sciencedirect.com/science/article/pii/S0303846720304753)
   **Authors**: B Aktar, B Balci, BD Colakoglu
   **Publication Info**: Clinical Neurology and Neurosurgery, 2020
   **DOI**: 10.1016/j.clineuro.2020.105654
9. **Title**: [**Clinical assessments in Parkinson's disease: scales and monitoring**](https://www.sciencedirect.com/science/article/pii/S0074774217300016)
   **Authors**: R Bhidayasiri, P Martinez-Martin
   **Publication Info**: International Review of Neurobiology, 2017
   **DOI**: 10.1016/bs.irn.2017.01.001

***Q2***: *Please provide many* ***more*** *academic papers (title, authors, DOI) referring to the following keywords:. "Parkinson disease " + "assessment scales" + "international classification of functioning" + "body" + "activity" + "participation”*

**Scholar ChatGPT said** (Talked to actions.sider.ai)


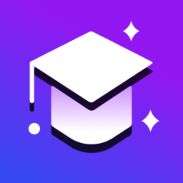


Here are **additional** academic papers related to Parkinson's disease, assessment scales, and the International Classification of Functioning (ICF):

1. **Title**: **[Identification of Common Problems for Parkinson Patients Using the International Classification of Function](https://www.researchgate.net/profile/Ilker-Celen/publication/337487472_monitoring_of_irrigation_schemes_using_by_termal_camera_mounted_UAVs/links/5ddbbf34299bf10c5a3162f1/monitoring-of-irrigation-schemes-using-by-termal-camera-mounted-UAVs.pdf#page=152)**
   **Authors**: Y Qin, Z Jiang, F Lin, D Xu, J Li, ZZ Chen
   **Publication Info**: FEB-FRESENIUS, 2019
   **DOI**: Not provided
2. **Title**: [**Functioning and Disability in Parkinson's Disease**](https://www.tandfonline.com/doi/abs/10.3109/09638288.2010.511688)
   **Authors**: A Raggi, M Leonardi, D Ajovalasit
   **Publication Info**: Disability and Rehabilitation, 2010
   **DOI**: 10.3109/09638288.2010.511688
3. **Title**: [**Factors Associated with Exercise Behavior in People with Parkinson Disease**](https://academic.oup.com/ptj/article-abstract/91/12/1838/2735195)
   **Authors**: T Ellis, JT Cavanaugh, GM Earhart
   **Publication Info**: Physical Therapy, 2011
   **DOI**: 10.2522/ptj.20100390
4. **Title**: [**Exploring Outcome Measures for Exercise Intervention in People with Parkinson's Disease**](https://onlinelibrary.wiley.com/doi/abs/10.1155/2013/572134)
   **Authors**: LA King, A Salarian, M Mancini, KC Priest
   **Publication Info**: Parkinson's Disease, 2013
   **DOI**: 10.1155/2013/572134
5. **Title**: [**Feasibility and Informativeness of the Patient-Specific Functional Scale with Patients with Parkinson's Disease**](https://www.tandfonline.com/doi/shareview/10.1080/09593985.2019.1571134)
   **Authors**: RW Bohannon, P Nair, M Green
   **Publication Info**: Physiotherapy Theory and Practice, 2020
   **DOI**: 10.1080/09593985.2019.1571134
6. **Title**: [**Measuring Participation as Defined by the International Classification of Functioning, Disability, and Health**](https://www.sciencedirect.com/science/article/pii/S0003999309001191)
   **Authors**: L Resnik, MA Plow
   **Publication Info**: Archives of Physical Medicine and Rehabilitation, 2009
   **DOI**: 10.1016/j.apmr.2009.02.009 DOI: [10.1016/j.apmr.2008.11.010](https://doi.org/10.1016/j.apmr.2008.11.010)
7. **Title**: [**Physical Activity in Patients with Parkinson's Disease: A Holistic Approach Based on the ICF Model**](https://www.sciencedirect.com/science/article/pii/S0303846720304753)
   **Authors**: B Aktar, B Balci, BD Colakoglu
   **Publication Info**: Clinical Neurology and Neurosurgery, 2020
   **DOI**: 10.1016/j.clineuro.2020.105654 DOI: [10.1016/j.clineuro.2020.106132](https://doi.org/10.1016/j.clineuro.2020.106132)
8. **Title**: [**Disability and Profiles of Functioning of Patients with Parkinson's Disease Described with ICF Classification**](https://journals.lww.com/intjrehabilres/FullText/2011/06000/Disability_and_profiles_of_functioning_of_patients.7.aspx)
   **Authors**: A Raggi, M Leonardi, D Ajovalasit
   **Publication Info**: International Journal of Rehabilitation Research, 2011
   **DOI**: Not provided
9. **Title**: [**Modernizing Daily Function Assessment in Parkinson's Disease Using Capacity, Perception, and Performance Measures**](https://movementdisorders.onlinelibrary.wiley.com/doi/abs/10.1002/mds.28377)
   **Authors**: W Maetzler, L Rochester, R Bhidayasiri
   **Publication Info**: Movement Disorders, 2021
   **DOI**: 10.1002/mds.28377
10. **Title**: [**Disability Rating Scales in Parkinson's Disease: Critique and Recommendations**](https://movementdisorders.onlinelibrary.wiley.com/doi/abs/10.1002/mds.26649)
    **Authors**: LM Shulman, M Armstrong, T Ellis
    **Publication Info**: Movement Disorders, 2016
    **DOI**: 10.1002/mds.26649

**The second (punctual) interrogation of Scholar ChatGPT**

***Q1:*** *Indicate academic papers (title, authors, DOI) referring to the following keywords: "Parkinson disease " + "assessment scales" + "international classification of functioning" + "body" + "activity" + "participation”*

**Scholar ChatGPT said**


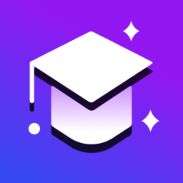


1. **Quantified measurement of activity provides insight into motor function and recovery in neurological disease**
   Authors: M. Busse, O. Pearson, R. V. Deursen, C. M. Wiles
   DOI: [10.1136/jnnp.2003.020180](https://dx.doi.org/10.1136/jnnp.2003.020180)
   [PDF URL](https://jnnp.bmj.com/content/jnnp/75/6/884.full.pdf)
2. **Motor, cognitive, and behavioral performance following unilateral ventroposterior pallidotomy for Parkinson disease**
   Authors: Donna Masterman, Antonio A. F. DeSalles, Robert W. Baloh, R. Frysinger, Dean J. Foti, Eric Behnke, C. Cabatan-Awang, Alexander Hoetzel, P. M. Intemann, Lynn A. Fairbanks, Jeff M. Bronstein
   DOI: [10.1001/ARCHNEUR.55.9.1201](https://dx.doi.org/10.1001/ARCHNEUR.55.9.1201)
3. **Effects of physical activity on functional readiness and quality of life in Parkinson’s disease patients**
   Authors: Hassan Reza Khalaji, Alireza Bahrami, Maryam Norouzian, Sadaf Mohammadzadeh
   DOI: [10.22038/MJMS.2014.3009](https://dx.doi.org/10.22038/MJMS.2014.3009)
4. **Comparison of the effect of balance exercises with and without space suits on the quality of life and motor performance of patients with Parkinson's disease**
   Authors: Majid Khodadadi, Nader Rahnama, Seyed Hossein Hashemi, Alireza Jahromi
   DOI: [10.22037/jrm.2017.1100285](https://dx.doi.org/10.22037/jrm.2017.1100285)
5. **Ambulatory monitoring provides a reliable measure of activity levels in neurological patients**
   Authors: M. Busse, O. Pearson, R. V. Deursen, C. M. Wiles
   DOI: [10.1136/jnnp.2003.020180](https://dx.doi.org/10.1136/jnnp.2003.020180)
   [PDF URL](https://jnnp.bmj.com/content/jnnp/75/6/884.full.pdf)

***Q2:*** *Indicate academic papers (title, authors, DOI) referring to the following keywords: Parkinson disease + "assessment scales" + "icf" + "body" + "activity" + "participation"*

**Scholar ChatGPT said**


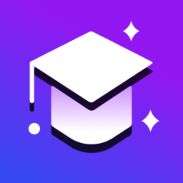


1. **Factors Associated With Exercise Behavior in People With Parkinson's Disease**
   Authors: T. Ellis, James T. Cavanaugh, G. Earhart, M. P. Ford, K. B. Foreman, L. Fredman, Jennifer K. Boudreau, Leland E. Dibble
   DOI: [10.2522/ptj.20100390](https://dx.doi.org/10.2522/ptj.20100390)
   [PDF URL](https://academic.oup.com/ptj/article-pdf/91/12/1838/17503502/ptj1838.pdf)
2. **Ambulatory Activity in People with Early Parkinson's Disease**
   Authors: Robyn M. Lamont, M. Morris, M. Woollacott, S. Brauer
   DOI: [10.1017/BrImp.2015.23](https://dx.doi.org/10.1017/BrImp.2015.23)
3. **Exercise capacity in people with Parkinson’s disease: which clinical characteristics are important?**
   Authors: H. Khalil, Reem Rehan, Alham Al-Sharman, Ala' S Aburub, M. Darabseh, M. Alomari, A. Aburub, K. El-Salem
   DOI: [10.1080/09593985.2022.2042634](https://dx.doi.org/10.1080/09593985.2022.2042634)
4. **The effect of physical activity on functional readiness and quality of life in people with Parkinson's disease**
   Authors: Hassan Reza Khalaji, Alireza Bahrami, Maryam Norouzian, Sadaf Mohammadzadeh
   DOI: [10.22038/MJMS.2014.3009](https://dx.doi.org/10.22038/MJMS.2014.3009)
5. **Self-efficacy and disability in community-dwelling people with Parkinson disease**
   Authors: T. Ellis, James T. Cavanaugh, G. Earhart, M. P. Ford, K. B. Foreman, L. Fredman, Jennifer K. Boudreau, Leland E. Dibble
   DOI: [10.2522/ptj.20100390](https://dx.doi.org/10.2522/ptj.20100390)

***Q3:*** *Indicate academic papers (title, authors, DOI) referring to the following keywords: "PD" + "assessment scales" + "international classification of functioning" + "body" + "activity" + "participation"*

**Scholar ChatGPT said**


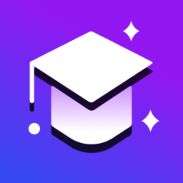


1. **Factors Associated With Exercise Behavior in People With Parkinson Disease**
   Authors: T. Ellis, James T. Cavanaugh, G. Earhart, M. P. Ford, K. B. Foreman, L. Fredman, Jennifer K. Boudreau, Leland E. Dibble
   DOI: [10.2522/ptj.20100390](https://dx.doi.org/10.2522/ptj.20100390)
   [PDF URL](https://academic.oup.com/ptj/article-pdf/91/12/1838/17503502/ptj1838.pdf)
2. **Long-term Effect of Regular Physical Activity and Exercise Habits in Patients With Early Parkinson Disease**
   Authors: Kazuto Tsukita, H. Sakamaki-Tsukita, Ryosuke Takahashi
   DOI: [10.1212/WNL.0000000000013218](https://dx.doi.org/10.1212/WNL.0000000000013218)
   [PDF URL](https://n.neurology.org/content/neurology/98/8/e859.full.pdf)
3. **Ataxia Rating Scales: Content Analysis by Linking to the International Classification of Functioning, Disability and Health**
   Authors: Mohammad Etoom, Alhadi M. Jahan, Alia A. Alghwiri, F. Lena, N. Modugno
   DOI: [10.3390/healthcare10122459](https://dx.doi.org/10.3390/healthcare10122459)
   [PDF URL](https://www.mdpi.com/2227-9032/10/12/2459/pdf?version=1670479861)
4. **Haemophilia item bank based on International Classification of Functioning, Disability and Health: a systematic review**
   Authors: Bao-Feng Geng, Chun Feng, Shou-guo Liu, Zhongli Jiang, F. Lin
   DOI: [10.1080/16078454.2023.2191077](https://dx.doi.org/10.1080/16078454.2023.2191077)
5. **Effect of aquatic exercise programs according to the International Classification of Functionality, Disability and Health domains in individuals with Parkinson’s disease: a systematic review and meta-analysis**
   Authors: Marcos Paulo Braz de Oliveira, Carla Rigo Lima, S. D. da Silva, Eluy Cristina Firmino Vaz Figueira, Brendon David Truax, S. Smaili
   DOI: [10.1080/09638288.2022.2164800](https://dx.doi.org/10.1080/09638288.2022.2164800)

***Q4:*** *Indicate academic papers (title, authors, DOI) referring to the following keywords: "PD" + "assessment scales" + "international classification of functioning" + "body" + "activity" + "participation"*

**Scholar ChatGPT said**


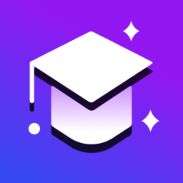


1. **Ataxia Rating Scales: Content Analysis by Linking to the International Classification of Functioning, Disability and Health**
   Authors: Mohammad Etoom, Alhadi M. Jahan, Alia A. Alghwiri, F. Lena, N. Modugno
   DOI: [10.3390/healthcare10122459](https://dx.doi.org/10.3390/healthcare10122459)
   [PDF URL](https://www.mdpi.com/2227-9032/10/12/2459/pdf?version=1670479861)
2. **Content comparison of four commonly used amputee mobility assessment scales in the literature by linking to the International Classification of Functioning, Disability, and Health**
   Authors: Yasin Ekinci, Ömer Faruk Yaşaroğlu, T. Düger
   DOI: [10.1097/PXR.0000000000000052](https://dx.doi.org/10.1097/PXR.0000000000000052)
3. **Effect of aquatic exercise programs according to the International Classification of Functionality, Disability and Health domains in individuals with Parkinson’s disease: a systematic review and meta-analysis**
   Authors: Marcos Paulo Braz de Oliveira, Carla Rigo Lima, S. D. da Silva, Eluy Cristina Firmino Vaz Figueira, Brendon David Truax, S. Smaili
   DOI: [10.1080/09638288.2022.2164800](https://dx.doi.org/10.1080/09638288.2022.2164800)
4. **Improved Functioning and Activity According to the International Classification of Functioning and Disability after Multidisciplinary Telerehabilitation for Post-COVID-19 Condition—A Randomized Control Study**
   Authors: Indre Bileviciute-Ljungar, J. Norrefalk, Kristian Borg
   DOI: [10.3390/jcm13040970](https://dx.doi.org/10.3390/jcm13040970)
   [PDF URL](https://www.mdpi.com/2077-0383/13/4/970/pdf?version=1707372954)
5. **COVID-19 pandemic and the international classification of functioning in multiple system atrophy: a cross-sectional, nationwide survey in Japan**
   Authors: K. Haruyama, M. Kawakami, I. Miyai, S. Nojiri, Toshiyuki Fujiwara
   DOI: [10.1038/s41598-022-18533-w](https://dx.doi.org/10.1038/s41598-022-18533-w)
   [PDF URL](https://www.nature.com/articles/s41598-022-18533-w.pdf)

**Q5:** *Indicate academic papers (title, authors, DOI) referring to the following keywords: "Parkinson disease " + "assessment instruments" + "international classification of functioning" + "body" + "activity" + "participation"*

**Scholar ChatGPT said**


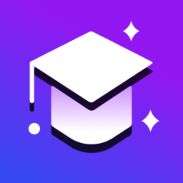


1. **Title**: "Evaluation of measures of upper limb functioning and disability in people with Parkinson disease: a systematic review"

**Authors**: EL Proud, KJ Miller, B Bilney, S Balachandran

**DOI**: 10.1016/j.apmr.2014.10.021

1. **Title**: "Health-related quality of life in patients with Parkinson's disease—a systematic review based on the ICF model"

**Authors**: JMT van Uem, J Marinus, C Canning

**DOI**: 10.1016/j.neubiorev.2015.12.004

1. **Title**: "Functional profile in Parkinson's disease and predictors of gait speed"

**Authors**: DD Iucksch, B Yamaguchi, LB Araújo

**DOI**: 10.1080/21679169.2022.2134449

1. **Title**: "Using the International Classification of Functioning, Disability and Health as a Framework to Examine the Association Between Falls and Clinical Assessment Tools"

**Authors**: M Beninato, LG Portney, PE Sullivan

**DOI**: 10.2522/ptj.20080214

1. **Title**: "Effect of resistance exercise on body structure and function, activity, and participation in individuals with Parkinson disease: a systematic review"

**Authors**: MPB de Oliveira, LM Dos Reis, ND Pereira

**DOI**: 10.1016/j.apmr.2021.02.011

***Q6:*** *Indicate academic papers (title, authors, DOI) referring to the following keywords: Parkinson disease " + "assessment instruments" + "icf" + "body" + "activity" + "participation"*

**Scholar ChatGPT said**


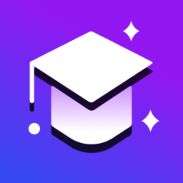


1. **Title**: "The International Classification of Functioning, Disability and Health as applied to Parkinson's disease: a literature review"

**Authors**: AS Vojciechowski, TGG Zotz, APC Loureiro

**DOI**: 10.4236/ojtr.2016.42003

1. **Title**: "Evaluation of measures of upper limb functioning and disability in people with Parkinson disease: a systematic review"

**Authors**: EL Proud, KJ Miller, B Bilney, S Balachandran

**DOI**: 10.1016/j.apmr.2014.10.021

1. **Title**: "Functional profile in Parkinson's disease and predictors of gait speed"

**Authors**: DD Iucksch, B Yamaguchi, LB Araújo

**DOI**: 10.1080/21679169.2022.2134449

1. **Title**: "Effect of resistance exercise on body structure and function, activity, and participation in individuals with Parkinson disease: a systematic review"
2. **Authors**: MPB de Oliveira, LM Dos Reis, ND Pereira

**DOI**: 10.1016/j.apmr.2021.02.011

1. **Title**: "Modernizing daily function assessment in Parkinson's disease using capacity, perception, and performance measures"

**Authors**: W Maetzler, L Rochester, R Bhidayasiri

**DOI**: 10.1002/mds.28377

***Q7:*** *Indicate academic papers (title, authors, DOI) referring to the following keywords: "PD" + "assessment instruments" + "international classification of functioning" + "body" + "activity" + "participation"*

**Scholar ChatGPT said**


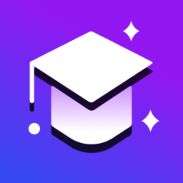


1. **Title**: "The International Classification of Functioning, Disability and Health as applied to Parkinson's disease: a literature review"

**Authors**: AS Vojciechowski, TGG Zotz, APC Loureiro

**DOI**: 10.4236/ojtr.2016.42003

1. **Title**: "Evaluation of measures of upper limb functioning and disability in people with Parkinson disease: a systematic review"

**Authors**: EL Proud, KJ Miller, B Bilney, S Balachandran

**DOI**: 10.1016/j.apmr.2014.10.021

1. **Title**: "Functional profile in Parkinson's disease and predictors of gait speed"

**Authors**: DD Iucksch, B Yamaguchi, LB Araújo

**DOI**: 10.1080/21679169.2022.2134449

1. **Title**: "Effect of resistance exercise on body structure and function, activity, and participation in individuals with Parkinson disease: a systematic review"

**Authors**: MPB de Oliveira, LM Dos Reis, ND Pereira

**DOI**: 10.1016/j.apmr.2021.02.011

1. **Title**: "Health-related quality of life in patients with Parkinson's disease—a systematic review based on the ICF model"

**Authors**: JMT van Uem, J Marinus, C Canning

**DOI**: 10.1016/j.neubiorev.2015.12.004

***Q8:*** *Indicate academic papers (title, authors, DOI) referring to the following keywords : "PD" + "assessment instruments" + "icf" + "body" + "activity" + "participation"*

**Scholar ChatGPT said**


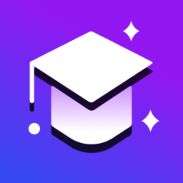


1. **Title**: "The International Classification of Functioning, Disability and Health as applied to Parkinson's disease: a literature review"

**Authors**: AS Vojciechowski, TGG Zotz, APC Loureiro

**DOI**: 10.4236/ojtr.2016.42003

1. **Title**: "Evaluation of measures of upper limb functioning and disability in people with Parkinson disease: a systematic review"

**Authors**: EL Proud, KJ Miller, B Bilney, S Balachandran

**DOI**: 10.1016/j.apmr.2014.10.021

1. **Title**: "Effect of resistance exercise on body structure and function, activity, and participation in individuals with Parkinson disease: a systematic review"

**Authors**: MPB de Oliveira, LM Dos Reis, ND Pereira

**DOI**: 10.1016/j.apmr.2021.02.011

1. **Title**: "Health-related quality of life in patients with Parkinson's disease—a systematic review based on the ICF model"

**Authors**: JMT van Uem, J Marinus, C Canning

**DOI**: 10.1016/j.neubiorev.2015.12.004

1. **Title**: "Functional profile in Parkinson's disease and predictors of gait speed"

**Authors**: DD Iucksch, B Yamaguchi, LB Araújo

**DOI**: 10.1080/21679169.2022.2134449

***Q9****:* Indicate academic papers (title, authors, DOI) referring to the following keywords: "Parkinson disease " + "assessment tools" + "international classification of functioning" + "body" + "activity" + "participation"

**Scholar ChatGPT said**


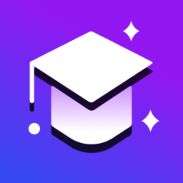


1. **Title**: "European physiotherapy guideline for Parkinson's disease"

**Authors**: S Keus, M Munneke, M Graziano

**DOI**: [10.1007/s00415-006-4008-0](https://www.parkinsonnet.nl/app/uploads/sites/3/2019/11/eu_guideline_parkinson_development_and_justification.pdf)

1. **Title**: "Evaluation of measures of upper limb functioning and disability in people with Parkinson disease: a systematic review"

**Authors**: EL Proud, KJ Miller, B Bilney, S Balachandran

**DOI**: 10.1016/j.apmr.2014.10.021

1. **Title**: "The effects of exercise on balance in persons with Parkinson's disease: a systematic review across the disability spectrum"

**Authors**: LE Dibble, O Addison, E Papa

**DOI**: 10.1097/NPT.0b013e318197f8e3

1. **Title**: "Using the International Classification of Functioning, Disability and Health as a Framework to Examine the Association Between Falls and Clinical Assessment Tools"

**Authors**: M Beninato, LG Portney, PE Sullivan

**DOI**: 10.2522/ptj.20080214

1. **Title**: "Effect of resistance exercise on body structure and function, activity, and participation in individuals with Parkinson disease: a systematic review"

**Authors**: MPB de Oliveira, LM Dos Reis, ND Pereira

**DOI**: 10.1016/j.apmr.2021.02.011

***Q10****: Indicate academic papers (title, authors, DOI) referring to the following keywords: "Parkinson disease " + "assessment tools" + "icf" + "body" + "activity" + "participation"*

**Scholar ChatGPT said**


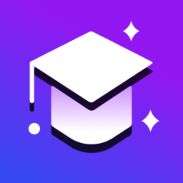


1. **Title**: "The International Classification of Functioning, Disability and Health as applied to Parkinson's disease: a literature review"

**Authors**: AS Vojciechowski, TGG Zotz, APC Loureiro

**DOI**: 10.4236/ojtr.2016.42003

1. **Title**: "Evaluation of measures of upper limb functioning and disability in people with Parkinson disease: a systematic review"

**Authors**: EL Proud, KJ Miller, B Bilney, S Balachandran

**DOI**: 10.1016/j.apmr.2014.10.021

1. **Title**: "Effect of resistance exercise on body structure and function, activity, and participation in individuals with Parkinson disease: a systematic review"

**Authors**: MPB de Oliveira, LM Dos Reis, ND Pereira

**DOI**: 10.1016/j.apmr.2021.02.011

1. **Title**: "Health-related quality of life in patients with Parkinson's disease—a systematic review based on the ICF model"

**Authors**: JMT van Uem, J Marinus, C Canning

**DOI**: 10.1016/j.neubiorev.2015.12.004

1. **Title**: "Functional profile in Parkinson's disease and predictors of gait speed"

**Authors**: DD Iucksch, B Yamaguchi, LB Araújo

**DOI**: 10.1080/21679169.2022.2134449

***Q11:*** *Indicate academic papers (title, authors, DOI) referring to the following keywords: "PD" + "assessment tools" + "international classification of functioning" + "body" + "activity" + "participation"*

**Scholar ChatGPT said**


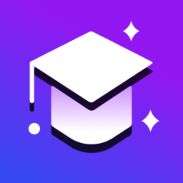


1. **Title**: "Evaluation of measures of upper limb functioning and disability in people with Parkinson disease: a systematic review"

**Authors**: EL Proud, KJ Miller, B Bilney, S Balachandran

**DOI**: 10.1016/j.apmr.2014.10.021

1. **Title**: "Functioning and disability in Parkinson's disease"

**Authors**: A Raggi, M Leonardi, D Ajovalasit

**DOI**: 10.3109/09638288.2010.511688

1. **Title**: "Health-related quality of life in patients with Parkinson's disease—a systematic review based on the ICF model"

**Authors**: JMT van Uem, J Marinus, C Canning

**DOI**: 10.1016/j.neubiorev.2015.12.004

1. **Title**: "Impairments, activity limitations, and participation restrictions of the International Classification of Functioning, Disability, and Health model in children with cerebral palsy"

**Authors**: A Mutlu, S Büğüsan, ÖK Kara

**DOI**: 10.4103/1817-1745.193365

1. **Title**: "Effect of resistance exercise on body structure and function, activity, and participation in individuals with Parkinson disease: a systematic review"

**Authors**: MPB de Oliveira, LM Dos Reis, ND Pereira

**DOI**: 10.1016/j.apmr.2021.02.011

***Q12****: Indicate academic papers (title, authors, DOI) referring to the following keywords: "PD" + "assessment tools" + "icf" + "body" + "activity" + "participation"*

**Scholar ChatGPT said**


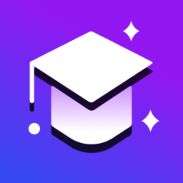


1. **Title**: "The International Classification of Functioning, Disability and Health as applied to Parkinson's disease: a literature review"

**Authors**: AS Vojciechowski, TGG Zotz, APC Loureiro

**DOI**: 10.4236/ojtr.2016.42003

1. **Title**: "Health-related quality of life in patients with Parkinson's disease—a systematic review based on the ICF model"

**Authors**: JMT van Uem, J Marinus, C Canning

**DOI**: 10.1016/j.neubiorev.2015.12.004

1. **Title**: "Effect of resistance exercise on body structure and function, activity, and participation in individuals with Parkinson disease: a systematic review"

**Authors**: MPB de Oliveira, LM Dos Reis, ND Pereira

**DOI**: 10.1016/j.apmr.2021.02.011

1. **Title**: "Evaluation of measures of upper limb functioning and disability in people with Parkinson disease: a systematic review"

**Authors**: EL Proud, KJ Miller, B Bilney, S Balachandran

**DOI**: 10.1016/j.apmr.2014.10.021

1. **Title**: "Functioning and disability in Parkinson's disease"

**Authors**: A Raggi, M Leonardi, D Ajovalasit

**DOI**: 10.3109/09638288.2010.511688

**Results:**

***From the provided results, no article meet the search criteria. The only 2 articles found were published out of the period within our PRISMA type systematic literature review and the other was not acces free.***
